# Functional map of arrestin binding to phosphorylated opsin, with and without agonist

**DOI:** 10.1038/srep28686

**Published:** 2016-06-28

**Authors:** Christian Peterhans, Ciara C. M. Lally, Martin K. Ostermaier, Martha E. Sommer, Jörg Standfuss

**Affiliations:** 1 Paul Scherrer Institute, Laboratory for Biomolecular Research, CH-5323, Villigen, Switzerland; 2 Institut für Medizinische Physik und Biophysik (CC2), Charité-Universitätsmedizin Berlin, Charitéplatz 1, D-10117, Berlin, Germany

## Abstract

Arrestins desensitize G protein-coupled receptors (GPCRs) and act as mediators of signalling. Here we investigated the interactions of arrestin-1 with two functionally distinct forms of the dim-light photoreceptor rhodopsin. Using unbiased scanning mutagenesis we probed the individual contribution of each arrestin residue to the interaction with the phosphorylated apo-receptor (Ops-P) and the agonist-bound form (Meta II-P). Disruption of the polar core or displacement of the C-tail strengthened binding to both receptor forms. In contrast, mutations of phosphate-binding residues (phosphosensors) suggest the phosphorylated receptor C-terminus binds arrestin differently for Meta II-P and Ops-P. Likewise, mutations within the inter-domain interface, variations in the receptor-binding loops and the C-edge of arrestin reveal different binding modes. In summary, our results indicate that arrestin-1 binding to Meta II-P and Ops-P is similarly dependent on arrestin activation, although the complexes formed with these two receptor forms are structurally distinct.

GPCRs mediate cellular signalling networks and regulate a wide variety of physiological and sensory responses. GPCRs are highly abundant in higher eukaryotes, especially in mammals, and approximately 1000 individual GPCR genes are known^1^,^2^. GPCR dysfunction and misfolding give rise to many diseases, and over 60% of prescribed drugs act on GPCRs^3^. The large and diverse GPCR superfamily is regulated by a small family of genetically and structurally conserved arrestin proteins. Arrestin-1 and -4 are solely expressed in photoreceptor neuron cells, whereas the two β-arrestins (arrestin-2 and -3) are ubiquitously expressed^4^. Arrestins both suppress G protein-mediated signalling as well as mediate their own signalling networks by scaffolding other signalling proteins. Recent studies report that different receptor phosphorylation patterns give rise to different conformations of arrestin, which are associated with different cellular functions^5^,^6^. The ability of different GPCR ligands to selectively elicit G protein-mediated or arrestin-mediated signalling, a phenomenon termed biased signalling, has been a topic of intense study and speculation^7^,^8^.

Structurally arrestins are composed of cup-shaped N- and C-domains and a long, flexible, auto-inhibitory C-tail. In the basal state, the C-tail is anchored to the N-domain by hydrophobic interactions within the 3-element interaction and by hydrogen bonding within the polar core. Activation of arrestin is triggered by binding to the active state of the receptor or the phosphorylated receptor C-terminus (Rpp). Based on mutagenesis studies^9^,^10^ (summarized in^11^) and the crystal structures of pre-activated arrestins^12^,^13^, the mechanism of arrestin activation is as follows: Binding of Rpp to phosphate-binding residues (phosphosensors) on β-strand I in the arrestin N-domain breaks the 3-element interaction, which releases the arrestin C-tail and thereby breaks the polar core. The displacement of the C-tail has widespread effects on the arrestin structure. The two domains of arrestin rotate against each other by ~20° and several loops in the central crest region are mobilized for receptor binding. The recent crystal structure of the complex of constitutively active human opsin fused to constitutively active mouse arrestin-1 (Ops^*^/arrestin-1) illustrates how these conformational changes facilitate coupling of arrestin to the helical bundle of the active receptor^14^.

This study focuses on arrestin-1, the rod visual arrestin, and its interactions with different functional forms of the GPCR rhodopsin. Rhodopsin consists of the apo-protein opsin and the Schiff base-linked inverse agonist 11-*cis* retinal. Light triggers isomerization of the ligand to the agonist all*-trans* retinal, which leads to formation of the active receptor species Metarhodopsin II (Meta II). Meta II couples to and activates the G protein transducin and is also phosphorylated by GPCR kinase-1 (GRK1), which enables arrestin binding^15^,^16^,^17^,^18^. About one minute after light-activation, phosphorylated Metarhodopsin II (Meta II-P) decays to opsin: the retinal Schiff base linkage spontaneously hydrolyses, and retinal exits the ligand binding pocket^19^. In its phosphorylated form, opsin is still bound by arrestin, and regeneration of the receptor with 11-*cis*-retinal is likely required to fully dissociate arrestin^20^,^21^,^22^. The interaction of arrestin with phosphorylated opsin (Ops-P) is functionally important, both to quench the residual activity of opsin, as well as to protect the rod cell in bright light by stimulating uptake of toxic all-*trans*-retinal into the pool of opsin^23^,^24^,^25^. Genetic pathologies that alter binding of arrestin-1 can lead to reduced vision by Oguchi disease^26^, congenital stationary night blindness^27^,^28^ or retinitis pigmentosa^29^.

Here we assess the functional contribution of each residue in arrestin-1 to binding of Ops-P, in comparison to Meta II-P. We used native rod outer segment (ROS) membranes, which preserve natural receptor density and phosphorylation patterns. Our results indicate both similarities and differences in how arrestin binds the two receptor forms. Site-directed fluorescence experiments suggest that some of these similarities, such as engagement of the finger loop, are due to the ability of Ops-P to adopt an active conformation similar to Meta II-P. Importantly, differences between the functional maps reflect different binding modes, which are distinct with respect to how the phosphorylated receptor C-terminus is bound within the arrestin N-domain, the extent of interdomain rotation, and deployment of the C-edge.

## Results

### Scanning mutagenesis of arrestin-1 and generation of functional maps for binding to Meta II-P and Ops-P

**Figure 1:**
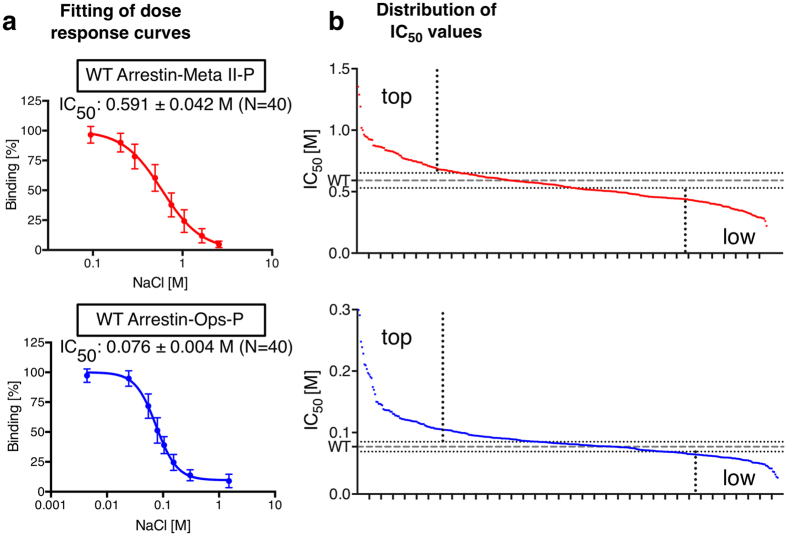
Quantification of arrestin mutants and calculation of IC_50_ values. Each alanine-mutant was expressed in *E. coli* and used for centrifugal pull-down analysis with native ROS membranes containing Meta II-P or Ops-P. Light-activated Meta II-P was assayed in parallel and simultaneously with the apo-form Ops-P. **(a)** NaCl titrations against arrestin-1 wild-type (WT) binding to Meta II-P *(red)* or Ops-P *(blue)* were used to calculate sigmoidal dose-response curves and extract IC_50_ values. **(b)** Distribution of IC_50_ values for the complete arrestin functional map when bound to phosphorylated Meta II-P *(red)* and Ops-P *(blue).* The dashed line indicates the WT IC_50_ values, and dotted lines indicate the doubled standard deviation calculated from arrestin WT data sets.

We applied alanine scanning mutagenesis on arrestin-1 to identify and compare the contributions of all side-chains in binding Meta II-P and Ops-P. Every residue in the arrestin-1 sequence, except the first methionine, was mutated to alanine, and native alanine residues were mutated to glycine^9^,^30^. Arrestin mutants were cloned in-frame with the fluorescent protein mCherry and expressed in *E. coli*. The fluorescence of mCherry facilitated the detection of the expression level and was used as an easy read-out to quantify the amount of arrestin pulled-down by ROS membranes in a high-throughput manner. The relative binding affinity of the arrestin mutants was assayed using titration of NaCl as multisite inhibitor (Fig. 1a). Mutations that increase or decrease binding strength resulted in higher or lower half maximum inhibitory concentrations of sodium chloride (IC_50_) compared to wild-type, respectively (Fig. 1b). We have previously established this experimental approach in order to generate a functional map of arrestin-1-binding to Meta II-P^9^.

**Figure 2:**
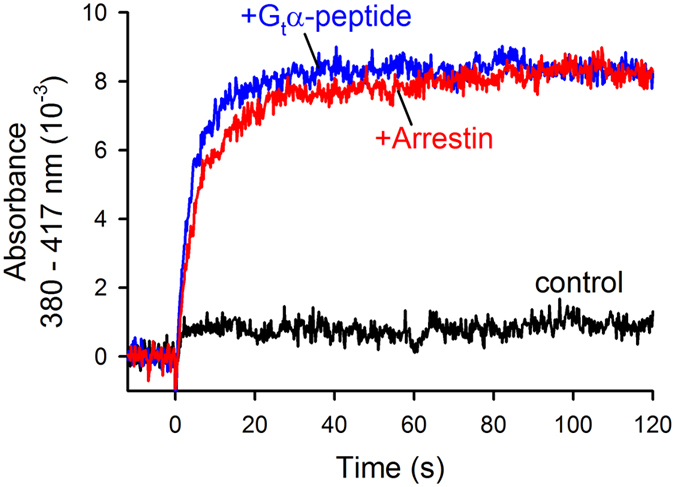
Functional test of receptor phosphorylation. The “Extra Meta II” assay measures the stabilisation of Meta II (λ_max_: 380 nm) over its precursor Meta I (λ_max_: 480 nm) by absorbance spectroscopy. In the absence of any binding partner, very little Meta II evolved (*black trace*). In the presence of the high-affinity analogue peptide derived from the C-terminus of the α-subunit of transducin (G_t_α-peptide, VLEDLKSCGLF, 350 μM), 100% of receptors were stabilised as Meta II (*blue trace*). In the presence of arrestin (10 μM), all receptors were also stabilised as Meta II (*red trace*). Hence, all receptors in our preparation were sufficiently phosphorylated to bind arrestin as Meta II-P. A high level of receptor phosphorylation is indicated by the fast rate of arrestin binding, which is comparable to G_t_α-peptide. This assay has previously been established as an indicator of functional receptor phosphorylation for studies of arrestin binding^22^.

Here we investigated arrestin-1 binding to highly phosphorylated opsin, Ops-P. ROS membranes were isolated from bovine retina and rhodopsin phosphorylation was carried out using the endogenous GRK1 according to a protocol that is optimized for high phosphorylation levels^22^. The phosphorylation level of ROS membranes was evaluated using arrestin-dependent Meta II-P stabilisation (Fig. 2). We prepared a single large batch of Ops-P, half of which was regenerated with 11-*cis*-retinal to form phosphorylated rhodopsin for the light-dependent formation of Meta II-P. Binding to Ops-P and Meta II-P were measured in parallel. In this way we generated two distinct functional maps of arrestin-1 binding to Ops-P and Meta II-P, which can be directly compared to one another.

The IC_50_ value derived from NaCl titration against wild-type arrestin binding to Meta II-P was determined in 40 independent measurements over the course of several months to be 0.59 ± 0.04 M, which is similar to previously reported values^9^,^22^,^31^ (Fig. 1a). This value is ~44% higher than that reported in our previous arrestin alanine scan study^9^, and we believe this difference is due to higher levels of receptor phosphorylation in the current study. We found that arrestin-1 binding to Ops-P was much more salt sensitive than Meta II-P binding (7-fold reduced IC_50_, 0.076 ± 0.004 M), which is consistent with previous reports^18^,^22^ (Fig. 1a). Salt titrations and pull-downs were performed for each arrestin-1 mutant, and wild-type was expressed and processed in parallel for each batch (see *Methods*). The dataset for Ops-P is 91% complete. Some mutants had to be rejected from the analysis due to poor expression or insufficient interaction with Ops-P membranes. For Meta II-P, the dataset is complete to 89%. A complete list of all IC_50_ values for Meta II-P and Ops-P can be found as Supplementary Table 1 information.

**Figure 3:**
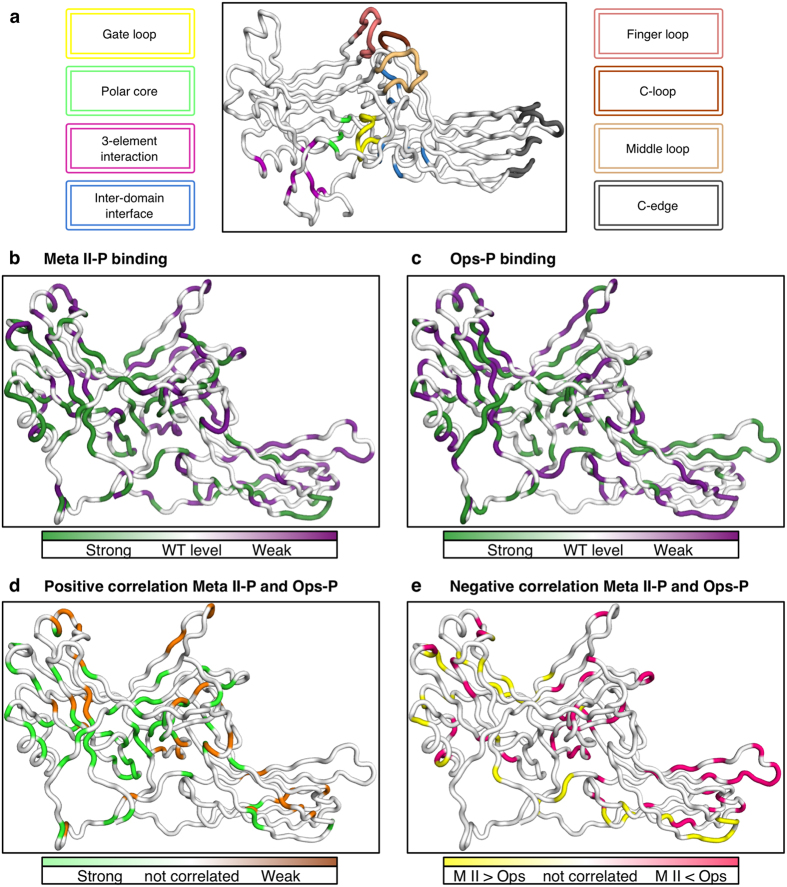
Functional maps of arrestin-1 binding to Meta II-P and Ops-P. (**a)** Important functional regions highlighted on the basal arrestin structure^32^. **(b)** IC_50_ values for Meta II-P binding plotted on the basal structure of arrestin-1. Mutations which significantly increased or decreased binding are coloured *green* and *purple*, respectively. **(c)** IC_50_ values for Ops-P binding. Colour code follows that described in (**a**). **(d)** Positive correlation of mutants for Meta II-P and Ops-P binding. Green – mutations which increased binding for both Meta II-P and Ops-P. *Orange* – mutations which decreased binding for both Meta II-P and Ops-P. **(e)** Negative correlation of mutants for Meta II-P and Ops-P binding. *Yellow* – mutations which increased affinity for Meta II-P but decreased affinity for Ops-P. *Pink* – mutations which decreased affinity for Meta II-P but increased affinity for Ops-P. For (**b**–**e**), only IC_50_ values outside the doubled standard deviation are shown.

We mapped the contributions of all side chains onto the arrestin structure^32^, using different colouring to indicate whether mutations increased or decreased binding strength (Fig. 3b,c). For both Ops-P and Meta II-P, mutations affecting binding strength positively as well as negatively are spread over the whole arrestin sequence (Fig. 3b,c), although many of the mutants with strong effects are clustered. 25% of tested residues outside the double standard deviation threshold are positively correlated, i.e. had the same general effect in both Ops-P and Meta II-P (Figs 1b and 3d). However, a similar number (24%) of residues are present as distinct clusters of negatively correlated residues (Fig. 3e). More information about calculation and ranking of IC_50_ values can be found in *Methods*.

### Functional map similarities

The polar core is a buried hydrogen bond network composed of residues from both the N- and C-domains, as well as the C-tail (D30, R175, D296, D303, T304, R382), which stabilises the basal state of arrestin^13^,^32^. Nearly all mutations in the polar core strongly increased binding to both Meta II-P and Ops-P (Table 1). The 3-element interaction also controls the arrestin activation state and involves hydrophobic side-chain interactions between β-strand I, α-helix I and the C-tail of arrestin (H10, V11, I12, F13, L107 L111, F375, V376, F377, F380)^33^. Disruption of the 3-element interaction and the release of the arrestin C-tail is crucial for arrestin activation and exposes a stretch of positively charged residues involved in Rpp binding^9^,^12^,^34^. Nearly all mutations within the 3-element interaction increased IC_50_ values for both Meta II-P and Ops-P binding (Table 1).

The finger loop (G68–S78) is a flexible loop in the arrestin central crest that is a critical receptor-binding element. Two recent crystal structures indicate that this loop binds in a near-helical conformation deep in the cytoplasmic crevice of the active receptor^14^,^35^. In our mutagenesis analysis, mutations in the finger loop decreased affinity to both Meta II-P and Ops-P (Table 1 and Fig. 3b–d). Residues 73, 74, 75, 77, 78 have low IC_50_ values for Meta II-P. For Ops-P residues 71, 73, 74, 76, 77, 78, 79 have weak binding when mutated. Similar to the finger loop, mutations on the 160-loop (H155–P165) generally decreased affinity to both Meta II-P and Ops-P (Fig. 3b–d).

**Figure 4:**
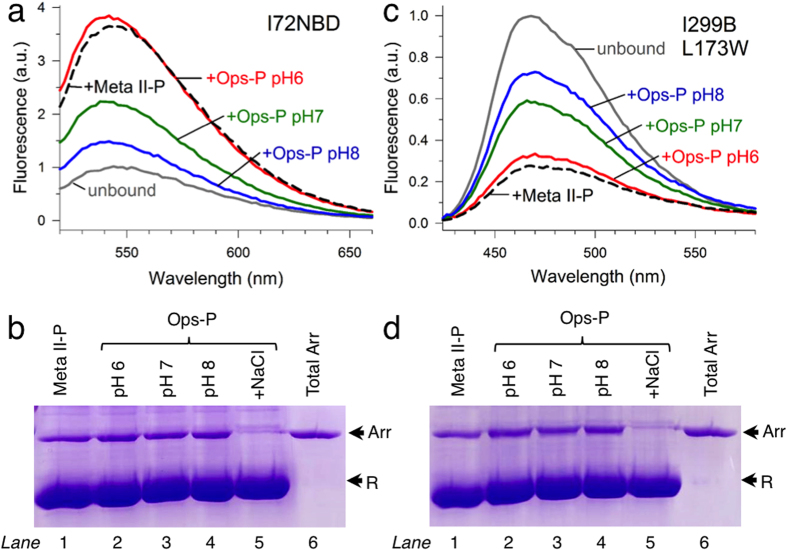
Site-directed fluorescence. (**a**) Steady-state fluorescence spectra of arrestin I72NBD (1 μM) were measured in the absence *(grey trace)* or presence of an excess of receptor (6 μM): light-activated Meta II-P at pH 7 *(dashed black trace)*, Ops-P at pH 6 *(red trace)*, Ops-P at pH 7 *(green trace)* or OpsP at pH 8 *(blue trace)*. (**b)** Centrifugal pull-down analysis of arrestin I72NBD binding to Meta II-P at pH 7 (lane 1) or Ops-P at pH 6, 7 or 8 (lanes 2, 3 and 4, respectively). As a negative control, no pull down of arrestin with Ops-P at pH 7 in the presence of 1 M NaCl was observed (lane 5). The total amount of arrestin in each pull-down experiment (4.5 μg) is shown in lane 6. The arrows indicate the location of arrestin (Arr) and receptor (R). (**c)** Same as described in (**a**), performed with arrestin I299B/L173W. (**d)** Same as described in (**b**), performed with arrestin I299B/L173W. Note that these experiments were performed in low-salt buffer in order to maximize arrestin binding to OpsP.

We were surprised to observe the importance of the finger loop for arrestin-1 binding to Ops-P, considering previous studies indicated that this loop is only engaged by the active receptor^22^,^36^,^37^,^38^,^39^. Thus we further characterized the interaction of Ops-P membranes with an arrestin-1 mutant labelled on the finger loop with the environmentally sensitive fluorophore *N,N′*-dimethyl-*N*-(iodoacetyl)-*N′*-(7-nitrobenz-2-oxa-1,3-diazol-4-yl)ethylenediamine (NBD). The fluorescence of the labelled arrestin mutant (I72NBD) increases dramatically when the finger loop is buried in the hydrophobic crevice of the active receptor^40^. Using an excess of receptor, we observed that Ops-P at pH 7 induced a fluorescence increase ~45% as great as that of arrestin I72NBD bound to Meta II-P (Fig. 4a). Interestingly, at pH 6 arrestin I72NBD binding to Ops-P resulted in a fluorescence signal comparable to that induced by Meta II-P. In contrast, arrestin binding to Ops-P at pH 8 resulted in a fluorescence increase only ~18% as great as Meta II-P. Centrifugal pull-down analysis employing the same samples described above indicated that all arrestin was membrane-bound in each case (Fig. 4b). Hence, the observed pH-dependent differences in I72NBD fluorescence in the presence of OpsP was not due to different levels of arrestin binding. We additionally examined the interaction of Ops-P with the arrestin mutant I299B/L173W, which is labelled with a bimane fluorophore on the gate loop (residues 296–305). In the basal unbound state, the gate loop of arrestin forms part of the intact polar core, and this mutant exhibits an enhanced fluorescence due to site 299 being buried by the C-tail. Upon arrestin binding to Meta II-P, the C-tail is displaced and the gate loop moves toward the N-domain, which brings site 299 into close proximity with the tryptophan residue at site 173^13^. This conformational change results in a significant quenching of the bimane fluorescence. Notably, this mutant is sensitive to the activation state of the receptor, and we previously reported that gate loop movement only occurs upon arrestin-1 coupling to Meta II-P and not inactive Ops-P^13^. In the present study, we observed that an excess of Ops-P at pH 7 induced gate loop movement in ~57% of the arrestin I299B/L173W (Fig. 4c). Similar to arrestin I72NBD, pH 6 favoured an Ops-P-dependent fluorescence change similar to that induced by Meta II-P, and pH 8 reduced the intensity of the fluorescence change to ~38% that of Meta II-P. Centrifugal pull-down analysis again confirmed that these differences in fluorescence were not due to different levels of arrestin binding (Fig. 4d). Together these results indicate that, depending on the pH, arrestin employs different binding modes when engaging Ops-P, which are distinct with respect to the finger and gate loops. In the *Discussion* we further elaborate on the molecular basis of these different binding modes, and the implications for understanding the functional maps.

### Functional map differences

Disruption of the 3-element interaction by mutation increased IC_50_ values for both Meta II-P and Ops-P binding. Interestingly alanine substitution of the bulky phenylalanine residues at sites 375 and 377, the main anchors of the C-tail, had a more dramatic effect on Ops-P compared to Meta II-P binding (~2.9-fold increase versus ~1.4-fold increase in IC_50_). Likewise, truncation of the arrestin C-tail (1–378, Δ379–404) had a more positive effect on Ops-P binding (4-fold increase in IC_50_) than Meta II-P binding (1.4-fold increase in IC_50_).

**Figure 5:**
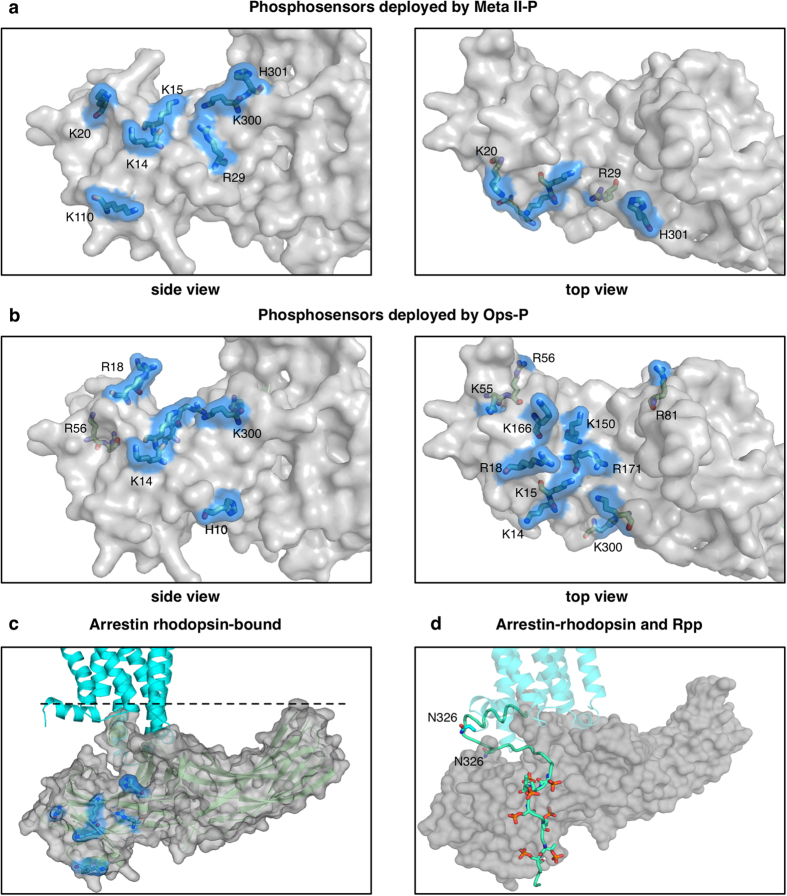
Different engagement of phosphosensors. Phosphosensing residues, whose mutation strongly decreased binding are highlighted in *blue* in (a) and (b) on pre-activated arrestin p44^13^. **(a)** Phosphosensitive residues deployed by Meta II-P (K14, K15, K20, R29, K110, K300 and H301) are located along the side of the arrestin N-domain. Side view (left) and top view (right) of arrestin are shown. The top view represents the orientation of arrestin as seen from the cytoplasmic face of the receptor. **(b)** Phosphosensing residues deployed by Ops-P (K14, K15, R18, K55, R56, R81, K150, K166, R171 and K300) are located in the cup of the arrestin N-domain. Views of arrestin are as in (**a**). **(c)** The phosphosensitive residues (*blue*) determined from the Meta II-P functional map are plotted on the Ops*/arrestin-1 complex^14^. The receptor is *cyan*, and the dashed line indicates the membrane plane. **(d)** The receptor phosphopeptide (Rpp) *(mint green)* aligned on arrestin. The Rpp model is adapted from^9^. N326, shown as sticks, is the last resolved residue in^14^ and is also shown as sticks in the Rpp model.

The N-domain of arrestin harbours many basic residues that could serve as phosphosensors, many of which have been implicated by mutagenesis^9^,^41^ and X-ray protein crystallography^12^,^13^. Conservation of identified phosphosensors in the different arrestin subtypes, especially arrestin-1 and arrestin-2, and their engagement in binding to conserved phosphorylation sites in different GPCR C-tails has been reviewed^8^. Direct comparison of the IC_50_ values for mutants of these potential phosphosensing sites reveals strikingly different patterns for Ops-P and Meta II-P binding (Table 1 and Fig. 5). Whereas K14 and K15 are important for both receptor forms, mutation of K5, K20, R29, K110, K300 and H301 most strongly affected Meta II-P binding. These sites line a positively charge cleft within the N-domain, which is exposed upon displacement of the C-tail. On the other hand Ops-P binding is was most affected by mutation of residues H10, R18, K55, R56, R81, K150, K166 and R171. These sites are mostly localized within the cup of the N-domain.

Several negatively correlated residues for Meta II-P and Ops-P binding in the arrestin N-domain reveal the differential engagement of phosphosensors (Fig. 3e). Significant clusters of negatively correlated residues are also present within the inter-domain interface and the C-edge. The inter-domain interface undergoes significant rearrangements upon arrestin activation, resulting in a ~20° twist of the arrestin C-domain relative to the N-domain^12^,^13^. Clusters of negatively correlated residues within the inter-domain interface include region 209–220 (Loop 12–13 near the inter-domain hinge), and region 310–324 (part of Loop17–18, which winds between the two domains of arrestin) (Table 1 and Fig. 3e). Notably, these areas are rearranged in pre-activated arrestin p44 as compared to basal arrestin-1. The root-mean-square deviation (RMSD) between basal and pre-activated arrestin p44 for region 209–220 is ~3.2 Å, and the RMSD for region 310–324 is ~4.6 Å. The rearrangements in these regions are directly related to the inter-domain rotation that accompanies arrestin activation^13^,^32^.

Besides the inter-domain interface, negatively correlated residues are clustered within the C-edge, specifically near the 270-loop (residues 268–274) and the 344-loop (residues 334–338) (Table 1 and Fig. 3e). Remarkably, the correlation between these two regions is exactly opposite: mutations within region 268–274 enhanced Meta II-P binding and decreased Ops-P binding, while mutations within 334–338 decreased Meta II-P binding and enhanced Ops-P binding. Similarly, mutations in the middle loop generally increased binding to Ops-P but had mixed effects on Meta-II. Mutations of residues 81–84 just adjacent to the finger loop region, on the other hand, increased affinity to Meta II-P but decreased affinity to Ops-P (Supplementary Table 1). Altogether the data thus indicates structurally similar but distinct binding modes of the two receptor forms.

### Structural context of functional maps

The first crystal structure of a GPCR in complex with arrestin was recently published^14^. This breakthrough was possible by fusing a constitutively active mutant form of human rhodopsin to both lysozyme (to facilitate crystallisation) and a constitutively active mutant form of mouse arrestin-1 (to favour arrestin binding to the receptor). The agonist all-*trans*-retinal is not resolved, yet the complex adopts an active conformation (Ops*) due to the activating mutations, presence of detergent, and low pH. The receptor-bound arrestin displays all the hallmarks of arrestin activation^12^,^13^, including displacement of the C-tail, breakage of the polar core, inter-domain rotation, and significant rearrangements of the central crest loops (e.g. finger, middle, C-loops).

**Figure 6:**
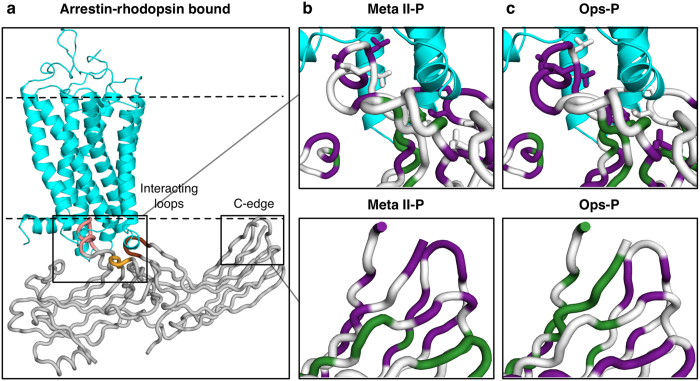
Mutational data on the Ops*/arrestin-1 complex structure. (**a)** Model derived from the arrestin-receptor complex structure^14^, rhodopsin *(cyan)*, and arrestin *(grey)*. Finger, middle and C-loop are coloured like in Fig. 3. Dashed lines indicate the membrane bilayer. **(b)** Arrestin-rhodopsin interaction coloured by IC_50_ values derived by Meta II-P interaction. **(c)** Arrestin-rhodopsin interaction coloured by IC_50_ values derived by Ops-P interaction. In (**b,c**), the receptor-arrestin interaction, coloured according to mutations which significantly increased *(green)* or decreased *(purple)* binding for Ops-P (colour code follows that from Fig. 3b,c). Core interaction residues as classified by EPPIC are shown as sticks^42^. Top panels in (**b,c**) show the interaction with loop 160, finger, middle and C-loop. Bottom panels show putative membrane interaction sites in the C-edge loops.

Arrestin makes several specific contacts with the receptor in the Ops*/arrestin-1 complex, and mutation of these contact points are expected to affect binding affinity. We analysed these binding interactions using the program EPPIC (Evolutionary Protein-Protein Interaction Classifier), which detects the core interacting residues in a complex and evaluates biocompatibility of the interface based on evolutionary conservation^42^. The computational analysis confirmed the interaction interface to be biologically relevant. Moreover, when our mutagenesis data for Meta II-P and Ops-P binding are plotted on the structure of the complex, it is clear how mutations in core interacting regions would interfere with coupling to the active receptor (Fig. 6).

Firstly, the finger loop is deeply inserted into the central cavity formed upon activation of the receptor. Mutation of several residues in the finger loop resulted in low IC_50_ values for Meta II-P (Fig. 6b), and Ops-P (Fig. 6c) binding. Differences in the mutation patterns are likely due to variations in engagement of the receptor (*see Discussion*), however weak binding of finger loop mutations on both Meta II-P and Ops-P reflects the biological relevance of finger loop interaction with the receptor.

Secondly, the 160-loop of arrestin contacts transmembrane helix 6 of the receptor, specifically via a hydrogen bond between D162 (D163 in mouse arrestin-1) and K245 on the receptor. Residues 161–163 are classified as rim residues by the program EPPIC. Rim amino acids directly interact with the receptor albeit their buried surface area is smaller than core residues. In agreement with this structural analysis, mutation of E161, D162 and K163 significantly decreases Meta II-P binding. The effect of the mutations is less pronounced in case of Ops-P, where D162 even resulted in slightly increased binding.

Thirdly, the C-loop within the central crest of arrestin makes hydrophobic contacts with intracellular loop (ICL2) of the receptor, which adopts a helical conformation. The importance of the contact point is reflected in the functional map of Meta II-P binding, where mutations in the C-loops lead to significantly reduced IC_50_ values.

The final contact point identified in the Ops*/arrestin-1 structure actually does not involve the receptor. The arrestin in the complex is bound to the receptor at an angle such that the 344-loop within the C-edge would be expected to interact with the membrane (although loop-344 is not completely resolved in the structure, and no membrane is present). Notably, mutation of several sites on the C-edge within the 344-loop and the nearby 200-loop significantly decrease binding to Meta II-P. Most of the influential mutations are hydrophobic or uncharged amino acids (e.g. L338, L339, L342, S345, F197, M198, S199), suggesting a possible role in interacting with the hydrophobic membrane interior. In contrast, mutation of sites within the 344-loop actually increased binding to Ops-P. Interestingly, mutations within the 270-loop adversely affected Ops-P binding, and many of these sites are polar or charged amino acids (e.g. E263, Q265, K267, N271). These differences suggest different roles for the C-edge loops in Meta II-P and Ops-P binding. Furthermore, the placement of the 344-loop within the putative membrane plane of the Ops*/arrestin-1 complex is supported by Meta II-P functional map.

Overall, we surmise that the Meta II-P functional data are most consistent with the Ops*/arrestin-1 complex structure, which would be compatible with the constitutively active state of the receptor in this complex.

## Discussion

This study was initiated based on the hypothesis that arrestin-1 employs different binding modes for Meta II-P and Ops-P^22^. The functional maps we present here indicate both similarities and differences regarding how arrestin-1 interacts with these two physiologically important forms of the receptor. In general the results demonstrate the structural and functional versatility that is common to all arrestin family members.

**Figure 7:**
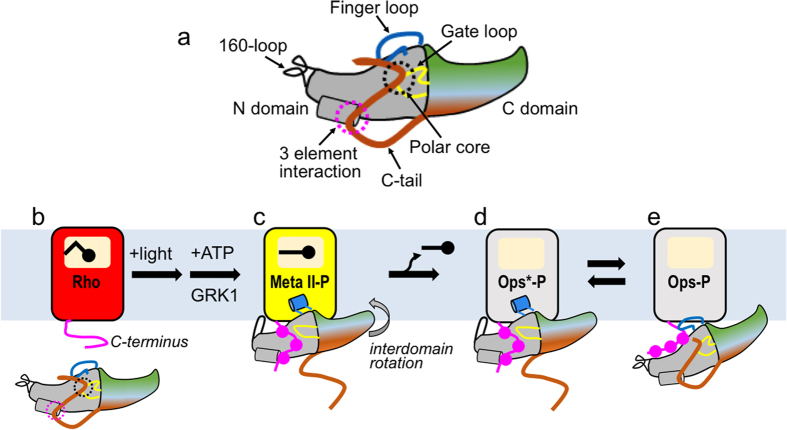
Interaction of arrestin with different functional forms of the receptor. **(a)** Cartoon representation of arrestin in the basal state. Notable loops include the finger loop (*blue*), the gate loop (*yellow*) and the 160-loop (*black*). The C-tail of arrestin is *dark orange* and interacts with the N-domain through the 3-element interaction and the polar core. **(b)** Dark state rhodopsin (Rho, *red*) and basal arrestin. **(c)** Light activation converts rhodopsin to Meta II, which is phosphorylated on the C-terminus by GRK1 and then bound by arrestin. Hallmarks of high affinity binding include receptor engagement of the finger loop and 160-loop, inter-domain rotation, and movement of the gate loop, which allows the phosphorylated receptor C-terminus to bind within a positively charged cleft in the N-domain. **(d,e)** Meta II-P decays to the apo-receptor Ops-P, which exists in a conformational equilibrium between an active (Ops*) and inactive (Ops) form. Arrestin binds Ops*-P similarly as Meta II-P. The interaction of arrestin with inactive Ops-P differs in the placement of the finger loop, the phosphorylated receptor C-terminus and how the C-edge is engaged.

The similarities in the functional maps we observed can be partially explained by the presence of active-state opsin in the pull-down experiments using Ops-P. In the native membrane, opsin exists in a pH-dependent conformational equilibrium between inactive opsin (Ops), which resembles dark-state rhodopsin, and active opsin (Ops*), which structurally resembles Meta II-P^43^,^44^,^45^,^46^,^47^. Vogel *et al*. reported a p*K* of the Ops/Ops* equilibrium of ~4, meaning that essentially no Ops* exists in native membranes at physiological pH. However, we surmise that receptor phosphorylation shifts the equilibrium toward Ops*, given that phosphorylation stabilises the active Meta II-P species over its inactive precursor Meta I^48^. The binding of arrestin would additionally influence the amount of receptor stabilised as Ops*-P^22^. For the current study, we estimated how much Ops-P could be stabilized in the active form by arrestin. In order to do this, we used fluorescently labelled arrestin mutants that report two key aspects of arrestin binding to the active receptor: finger loop engagement (I72NBD) and gate loop movement (I299B/L173W) (Fig. 4a,c). We used a six-fold excess of receptor to arrestin, which is a reasonable approximation of the conditions used in the pull-downs employing arrestin-mCherry mutants (see *Methods*). The fluorescence data indicate that about half of the 1 μM arrestin was bound to the active receptor form Ops*-P at pH 7, since the fluorescence signal obtained with Ops-P at pH 7 was about half as great as that obtained with Meta II-P (Fig. 4a,c). This result means that about 10% of the total opsin was stabilised as Ops*-P in complex with arrestin (0.5 μM / 6 μM = 0.083). Furthermore, we observed a pH-dependence in the fluorescence signals indicative of arrestin binding to the active receptor (Fig. 4a,c). Arrestin binding to Ops-P at pH 6 was similar to Meta II-P, and pH 8 significantly decreased the amount of Ops-P bound in the active form. Given that Ops*-P is favoured at lower pH values^43^, these observations suggest arrestin binds Ops*-P similarly as Meta II-P, at least with regard to the finger and gate loop. Despite these similarities, Meta II-P and Ops*P binding are biochemically distinct with respect to salt sensitivity. The difference can be explained by the coupling between the [Ops-P ↔ Ops*-P] and the [Arr + Ops*-P ↔ Arr●Ops*-P] equilibria. This coupling lowers the apparent affinity of arrestin for Ops*-P, because the conformational equilibrium of the receptor is heavily shifted toward inactive Ops-P in the native membrane. The fluorescence experiments presented here suggest that arrestin binding shifts the receptor conformational equilibrium by stabilizing Ops*-P (Fig. 7).

Based on the fluorescence experiments, we conclude that our functional map of Ops-P binding represents a combination of Ops-P and Ops*-P characteristics. The complex of arrestin with Ops*-P is structurally very similar to that with Meta II-P. In contrast, arrestin binding to inactive Ops-P is primarily electrostatic, and the finger loop is not engaged nor is the gate loop displaced (Fig. 7)^13^,^18^,^22^. Here we discuss the similarities and differences between the functional maps, keeping in mind the findings from the fluorescence experiments. We attempt to differentiate between arrestin elements important for binding inactive Ops-P and active Ops*-P.

The Meta II-P and Ops-P functional maps are very similar with respect to the key structural elements controlling phosphorylation-dependent arrestin activation: the polar core and the C-tail. These two elements stabilise the inactive, basal state of arrestin and their disruption by mutagenesis has long been known to lead to constitutive activity in arrestin^31^,^32^,^49^,^50^. Recent crystal structures of C-terminally truncated arrestin^13^,^51^ and the R175E arrestin mutant^52^ indicate how these mutations structurally activate arrestin for receptor binding. Alanine substitutions in the polar core or 3-element interaction increased arrestin binding to both Meta II-P and Ops-P. These mutations pre-activate arrestin and lower the energy barrier for coupling to the active receptor^53^. Hence, we hypothesize that pre-activated mutants are able to stabilize Ops*-P to a greater extent, which would explain the striking similarities between the Meta II-P and Ops-P functional maps for the polar core and 3-element interaction.

Interestingly, mutations which displaced the C-tail increased affinity for Ops-P to a greater extent than Meta II-P. These mutations included alanine substitution of the bulky hydrophobic residues involved in anchoring the C-tail within the 3-element interaction (F375 and F377), and C-tail truncation. C-tail displacement exposes a large area of electropositive surface within the arrestin N-domain^13^,^51^. Given the fact that arrestin binding to Ops-P is primarily electrostatic and heavily dependent on the level of receptor phosphorylation, it is not surprising that C-tail displacement would dramatically increase affinity for Ops-P^18^,^22^. A similar effect is observed for p44, a naturally occurring splice variant of arrestin-1 that lacks the C-tail, which has significantly enhanced affinity for all forms of phosphorylated rhodopsin, even inactive dark-state rhodopsin and Ops-P^10^.

Meta II-P and Ops-P binding were both negatively affected by mutations in the finger loop and 160-loop, which engage the active receptor in the Ops*/arrestin-1 complex^14^, this similarity could suggest a common binding mode, although it is also possible that these loops engage Meta II-P and Ops-P differently. Both of these loops are intrinsically very flexible and adopt a variety of conformations, or are not visible in the many crystal structures of arrestin^13^,^32^,^51^,^54^,^55^,^56^. Notably, the positioning of the finger loop in the Ops*/arrestin-1 complex and the complex of Ops* with a peptide analogue of the finger loop are different^14^,^35^. This difference could be simply due to the different structural constraints (free peptide versus loop anchored to the body of arrestin) or the mutations introduced into opsin in the Ops*/arrestin-1 complex. Yet it is also possible that the flexible finger loop can adopt a variety of conformations within the cytoplasmic crevice of the active receptor, which is supported by DEER studies of arrestin-1 binding to rhodopsin^57^. Different finger loop binding modes for Meta II-P (or Ops*-P) and Ops-P could also be implied by the opposite mutagenesis patterns seen in the residues 81–84, adjacent to the finger loop region.

Despite the many similarities between the Meta II-P and Ops-P functional maps, we detected significant differences that could indicate distinct binding modes. Firstly, the mutation of potential phosphosensors within the arrestin N-domain, in the region where the phosphopeptide derived from the V2 vasopressin receptor interacts with arrestin-2^12^ had dramatically distinct effects on Meta II-P and Ops-P binding^13^,^41^. A plot of affected residues on the arrestin surface yields distinct “phosphosensing footprints” for Meta II-P and Ops-P (Fig. 5). Meta II-P binding was most negatively affected by mutation of residues which line the positively-charged cleft within the N-domain, which is exposed upon full displacement of the C-tail^12^,^13^. In contrast, Ops-P binding was most affected by mutation of residues within the cup of the N-domain. Surprisingly, mutation of these residues had exactly the opposite effect on Meta II-P binding. This difference indicates that these residues are playing different roles in Ops-P and Meta II-P binding. In the case of Ops-P, we hypothesize that these residues bind Rpp. In the case of Meta II-P, these residues are in close proximity to the cytoplasmic face of the receptor, and their mutation would make the N-domain cup more electronegative. This change would increase binding affinity to Meta II-P, since the cytoplasmic face of the receptor is strongly electropositive^14^.

Overall these results indicate that Rpp binds arrestin differently for Meta II-P and Ops-P. We believe the arrestin-2/V2Rpp structure^12^ is similar to how Rpp binds in the arrestin/Meta II-P complex, although some differences likely exist because of the different phosphorylation patterns between the V2R and rhodopsin^8^. Distinct receptor phosphorylation patterns, which arise from ligand bias and/or GPCR C-tail subtype, are believed to engage specific clusters of phosphosensors and thereby direct the cellular functions of arrestin^5^,^6^,^8^. However, here we observe different phosphosensor engagement, depending on the presence or absence of agonist for the same Rpp phosphorylation pattern. In contrast to the arrestin/Meta II-P complex, we speculate that Rpp binds within the cup of the N-domain in the arrestin-1/Ops-P complex (Fig. 5). These different modes in Rpp binding are supported by site-directed fluorescence experiments, which indicated that the arrestin gate loop is displaced from its basal position only in complex with Meta II-P^22^. Movement of the gate loop is necessary for Rpp to access the positively-charged cleft within the N-domain. It is interesting to note the correlation between the mutation patterns in our functional maps and the different phosphorylation requirements of Meta II-P and Ops-P binding. Only four residues (K5, K14, R29, H301) strongly affected Meta II-P binding, which corresponds well to two to three phosphates that are required for maximal Meta II-P binding^17^,^18^. In contrast, nine residues strongly affected Ops-P binding (belong to the group of the lowest 20%), which mirrors the dependence of Ops-P binding on high levels of receptor phosphorylation^18^,^22^.

The Meta II-P and Ops-P functional maps are also distinct with respect to the inter-domain interface. For example, mutation of residues within region 310–324 of Loop17–18, increased Ops-P binding and weakened Meta II-P binding. Most of the critical sites are aliphatic residues (e.g. I311, G315, I316, I323, L324) that are solvent-exposed in the basal state structure and more buried in the pre-active p44 structure^13^. We hypothesize that mutation of these residues may interfere with the hydrophobic packing necessary to stabilise the new inter-domain orientation of the arrestin active state. Hence, the inter-domain rotation seen in the pre-active arrestin structures might be more critical for Meta II-P binding, and Ops-P binding might be different in this respect.

The rearrangement of the inter-domain interface causes a rotation of the two domains of arrestin against one another. In the Ops*/arrestin-1 complex structure^14^ the asymmetric binding of arrestin to the receptor, along with the twist of the C-domain, positions the loops of the C-edge within or near the theoretical plane of the membrane adjacent to the receptor. Previous site-directed fluorescence studies suggested the C-edge might interact with the membrane, or another receptor in a one-to-two complex^22^. Intriguingly, we observed strong changes in IC_50_ values, both positive and negative, when side chains in the C-edge were removed by alanine substitution. Strong and weak binders are clustered in a directly reversed manner between Meta II-P and Ops-P. Although the function of the C-edge is currently not well understood, the different patterns seen between Meta II-P and Ops-P binding could reflect distinct modes in membrane binding or different receptor binding stoichiometries. Considering the orientation of the C-edge is controlled by the extent of inter-domain rotation, it is likely that the positive and negative correlations seen in the inter-domain interface and in the C-edge are related and reflect distinct binding modes employed by arrestin for Ops-P and Meta II-P.

## Conclusions and Outlook

The functional maps we present here indicate both similarities and differences in how arrestin binds the phosphorylated apo-receptor Ops-P and the phosphorylated agonist-activated receptor Meta II-P.

To date rhodopsin is the only GPCR for which we have a crystal structure in complex with arrestin. Determination of this structure was only possible through heavy engineering of both rhodopsin and arrestin, which could significantly alter the complex. Our functional maps were obtained under physiological conditions and in a native membrane environment. The Meta II-P functional map confirms the core interaction sites seen in the structure and indicates how the phosphorylated receptor C-terminus, which is absent in the structure, binds arrestin. Importantly, comparison of the Meta II-P and Ops-P functional maps indicates a versatility in the way that GPCRs are bound by arrestin. Arrestins have a variety of functions, including desensitization of G protein signalling, receptor internalization and arrestin-mediated signalling. The known modulators of these arrestin functions, such as biased agonists that stabilize different active receptor conformations or stimulate different receptor phosphorylation patterns, could reasonably result in different conformations of receptor-bound arrestin and/or different receptor binding modes. Scanning mutagenesis may provide a way to isolate the functional footprints that orchestrate the complexity of GPCR and arrestin signalling.

## Methods

### Receptor preparations

Rod outer segment disc membranes (ROS) were isolated from frozen bovine retina (W.K. Lawson Company, USA), and phosphorylation was carried out using the native rhodopsin kinase present in the ROS, exactly as previously described^22^. Receptor phosphorylation was quenched with 20 mM hydroxylamine, and subsequent washes removed the hydroxylamine and peripheral ROS proteins (e.g. native arrestin). Phosphorylated rhodopsin (Rho-P) was prepared by the addition of a 3-fold molar excess of 11-*cis*-retinal to Ops-P under dim red light. The 11-*cis*-retinal was generated from commercially available all-*trans*-retinal (Sigma-Aldrich) and purified by high-pressure liquid chromatography. For the current study, a single large-scale preparation of Ops-P and Rho-P was made from 400 retinas (total yield of 240 mg of receptor), so that all arrestin mutants were tested using the same batch of phosphorylated ROS. The phosphorylation level of the preparation was assessed by the Extra Meta II assay, which is described in detail elsewhere^22^,^40^.

### Arrestin expression and cell lysate preparation

Detailed information concerning the constructs used in this study has been previously presented^9^,^30^. In brief, bovine arrestin-1 (SAG) was cloned into the “EgWoMiPi” vector suitable for mammalian and bacterial expression. The fluorescent protein “mCherry” was connected by a short linker sequence (GSSG) and a TEV protease cleavage site (ENLYFQGS) to the C-terminal part of the expression cassette. Mutations were introduced by site-directed PCR using the program AAscan^30^. Mutant construct plasmids were used to transform chemically competent *E.coli* BL21(DE3) cells (Stratagene). Transformed cells were grown as previously described^9^. Briefly, cells were grown at 37 °C, and expression of arrestin-mCherry was induced by 100 μM IPTG at an OD of 0.6. Expression in 200 mL LB medium was carried out at 20 °C for 18 h. Wild-type arrestin was always expressed alongside the mutants for each data set. Cells were harvested by centrifugation, and pellets were frozen in liquid nitrogen and stored at -80 °C. Frozen cell pellets were thawed on ice and resuspended in 1.8 mL of lysis buffer (10 mM HEPES pH 7.0, 10 mM NaCl, 0.1 mM EDTA, 5 mM DTT) containing protease inhibitor cocktail (Roche) and PMSF. For cell lysates destined for pull-down experiments using Ops-P, lysis buffer additionally contained 5 mM hydroxylamine and 10 mM NaCl. For cell lysates destined for pull-down experiments using Meta II-P, lysis buffer additionally contained 1 mM MgCl_2_, and 100 mM NaCl. Lysozyme (0.2 mg/mL) and DNAase (20 μg/mL) were added to the resuspended cells, which were lysed by sonication. The lysate was cleared by centrifugation (13800 × *g*, 90 min, 4 °C).

### Centrifugal pull-down assay

Cell lysate containing expressed arrestins-mCherry fusion proteins were assayed similarly as before^9^. Briefly, arrestin mutants and wild-type control were analysed in parallel using a 96-well centrifugal pull-down assay. For each arrestin construct, cleared cell lysate containing arrestin-mCherry (800 μL) was mixed with ROS membranes (100 μl) containing 135 μg of Ops-P or phosphorylated rhodopsin. This “mastermix” was divided into 8 wells (100 μl each) on a 96-well plate. The wells were pre-loaded with 100 μL of buffer containing increasing NaCl concentrations. Final NaCl concentrations for Meta II-P pull-downs were 50, 159, 249, 450, 707, 999, 1600, 2500 mM and final NaCl concentrations for Ops-P pull-downs were 4.4, 24, 54, 79, 104, 154, 304, 1504 mM. The final concentration of receptor was 1.85 μM and the final concentration of arrestin was estimated at 0.1–0.6 μM based on mCherry fluorescence. Plates were incubated at 37 °C for 2 min and then illuminated with bright light and immediately centrifuged at 6168 × *g* for 20 min at 4 °C. The membrane pellets were washed two times with buffer.

### Fluorescent mCherry detection and dose-response curve fitting

Washed membrane pellets were resupended in 100 μL buffer (10 mM HEPES pH 7.0, 10 mM NaCl, 0.1 mM EDTA, 5 mM DTT). Buffer for Meta II-P samples additionally contained 1 mM MgCl_2_ and 100 mM NaCl. Samples were transferred to new plates for fluorescence detection, and mCherry fluorescence was measured using a microplate reader (λex = 488 nm, λem = 612 nm). Fluorescence signals were normalised for each mutant, such that the highest signal equalled 100%. Dose-response curve fitting was performed with GraphPad Prism using a symmetrical sigmoidal distribution with variable slope (four-parameter dose response fit) using constraints for top and bottom values [Y = Bottom + (Top − Bottom)/(1 + 10^((LogIC50-X)*HillSlope))]. Half-maximum inhibitory concentrations (IC_50_) were thus obtained for all mutants for Ops-P and Meta II-P binding.

### Analysis of IC_50_ values

For the two datasets derived from Ops-P and Meta II-P binding, IC_50_ values were sorted from top to bottom for the entire dataset. Mutant IC_50_ values inside the double-standard deviation calculated from 40 wild-type measurements were not considered significantly different than wild-type and have been rejected from analysis. The significant values were grouped and ranked according to top 20%, top 30%, low 30%, and low 20% within the sorted datasets. The functional maps were generated by plotting the ranked residues onto the arrestin structure using distinct colouring for top and low IC_50_ values (Fig. 3b,c). Mutations which resulted in significantly higher or lower IC_50_ values for both Ops-P and Meta II-P binding were considered positively correlated. Mutations which resulted in a high IC_50_ value for one dataset and a low IC_50_ value for the other were considered negatively correlated. Positive and negative correlations were also plotted onto the arrestin structure (Fig. 3d,e).

### Site-directed fluorescence experiments

Recombinant bovine arrestin-1 mutants (C63A, C128S, C143A, W194F, I72C and C63A, C128S, C143A, W194F, L173W, I299C) were expressed and purified from *E. coli* as previously described^58^. The I72C mutant was labelled with IANBD, and the L173W/I299C mutant was labelled with monobromobimane (fluorophores purchased from ThermoFisher Scientific), and steady-state fluorescence was measured using a SPEX Fluorolog (1680) instrument as reported before^40^. The NBD fluorophore was excited at 500 nm, and the bimane fluorophore was excited at 390 nm. In general, the fluorescence of 1 μM labelled arrestin mutant was measured in the absence or presence of a six-fold excess of Rho-P or Ops-P at 20 °C. Rho-P was converted to Meta II-P using bright light (>495 nm, 10 s), and the fluorescence of the labelled arrestin was measured immediately after light-activation of the sample. Samples were suspended in 50 mM HEPES at pH 6, 7, or 8. Fluorescence spectra were plotted and analysed in the graphing program Sigma Plot, and the spectra in Fig. 4 are normalized to the spectrum of unbound arrestin. Complementary pull-down experiments using identical samples used for fluorescence experiments were performed as described^13^. After centrifugation, membrane pellets were solubilized in SDS loading buffer and subjected to SDS-PAGE (12%). Gels were stained with Coomassie brilliant blue, destained, and imaged using a scanning device.

## Additional Information

**How to cite this article**: Peterhans, C. *et al*. Functional map of arrestin binding to phosphorylated opsin, with and without agonist. *Sci. Rep.* **6**, 28686; doi: 10.1038/srep28686 (2016).

## Supplementary Material

Supplementary Information

## Figures and Tables

**Table 1:**
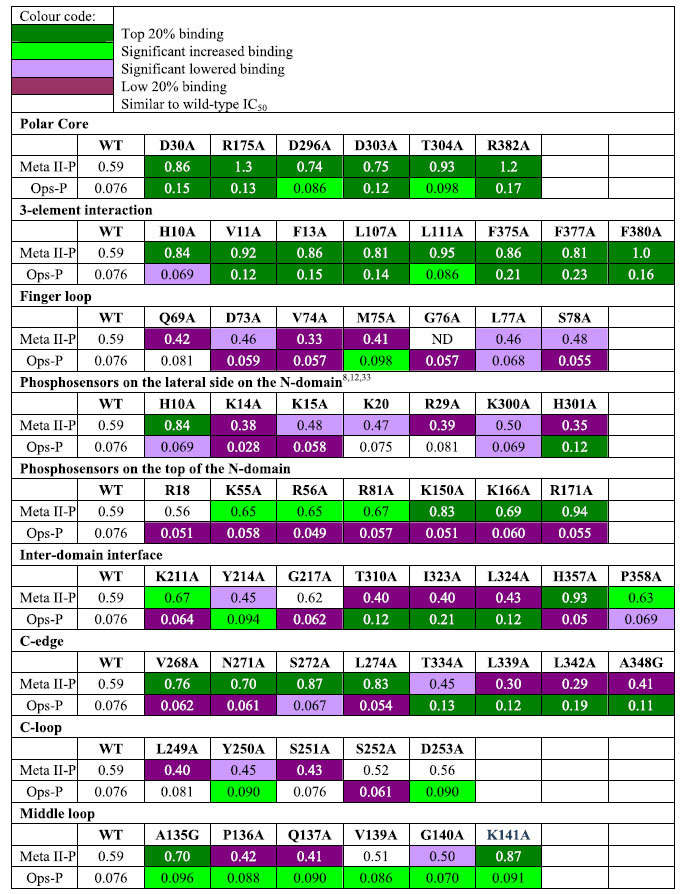
Selection of IC_50_ values in functionally important regions of arrestin-1.
