# Corrigendum: Functional map of arrestin binding to phosphorylated opsin, with and without agonist

**DOI:** 10.1038/srep30224

**Published:** 2016-07-22

**Authors:** Christian Peterhans, Ciara C. M. Lally, Martin K. Ostermaier, Martha E. Sommer, Jörg Standfuss

Scientific Reports 6: Article number: 2868610.1038/srep28686; published online: 06 28 2016; updated: 07 22 2016

In this Article, Martin K. Ostermaier is incorrectly listed as being affiliated with ‘Institut für Medizinische Physik und Biophysik (CC2), Charité-Universitätsmedizin Berlin, Charitéplatz 1, D-10117, Berlin, Germany’. The correct affiliation is listed below:

Paul Scherrer Institute, Laboratory for Biomolecular Research, CH-5323, Villigen, Switzerland

