# Supplementary material for: Functional map of arrestin binding to phosphorylated opsin, with and without agonist
Source: Sci Rep. 2016 Jun 28;6:28686. doi: 10.1038/srep28686 (PMC4923902; doi:10.1038/srep28686)
Supplement: Supplementary Information [file srep28686-s1.doc]

**Functional map of arrestin binding to phosphorylated opsin, with and without agonist**

Christian Peterhans^1^, Ciara Lally^2^, Martin K. Ostermaier^1^, Martha E. Sommer^2^ & Jörg Standfuss^1^

to whom correspondence should be addressed:

Jörg Standfuss

joerg.standfuss@psi.ch

Martha E. Sommer

martha.sommer@charite.de

^1^ Paul Scherrer Institute, Laboratory for Biomolecular Research, CH-5323, Villigen

^2^ Institut für Medizinische Physik und Biophysik (CC2), Charité-Universitätsmedizin Berlin, Charitéplatz 1, D-10117, Berlin, Germany

| **Meta II-P IC_50_** | | **N** | **R^2^** |  | **Ops-P IC_50_** | | **N** | **R^2^** |
| --- | --- | --- | --- | --- | --- | --- | --- | --- |
| K2A | 0.7931 | 1 | 0.9199 |  | K2A | 0.07994 | 1 | 0.9825 |
| A3G | 0.4925 | 1 | 0.9064 |  | A3G | 0.11380 | 1 | 0.9312 |
| N4A | 0.8664 | 1 | 0.9931 |  | N4A | 0.07617 | 1 | 0.9884 |
| K5A | 0.3365 | 1 | 0.9833 |  | K5A |  |  |  |
| P6A | 0.7178 | 1 | 0.9702 |  | P6A | 0.12110 | 1 | 0.9807 |
| A7G |  |  |  |  | A7G |  |  |  |
| P8A |  |  |  |  | P8A |  |  |  |
| N9A |  |  |  |  | N9A | 0.07581 | 1 | 0.9578 |
| H10A | 0.8373 | 1 | 0.8319 |  | H10A | 0.06884 | 1 | 0.8359 |
| V11A | 0.9228 | 1 | 0.9237 |  | V11A | 0.11820 | 2 | 0.9710 |
| I12A | 0.8628 | 3 | 0.9825 |  | I12A | 0.11890 | 2 | 0.9996 |
| F13A | 0.8628 | 3 | 0.8200 |  | F13A | 0.14650 | 2 | 0.9787 |
| K14A | 0.3763 | 3 | 0.9872 |  | K14A | 0.02805 | 2 | 0.9249 |
| K15A | 0.4829 | 3 | 0.9873 |  | K15A | 0.05822 | 2 | 0.9757 |
| I16A | 0.5937 | 2 | 0.8538 |  | I16A | 0.10040 | 1 | 0.9794 |
| S17A | 0.4814 | 2 | 0.9096 |  | S17A | 0.14580 | 1 | 0.9364 |
| R18A | 0.5581 | 2 | 0.9801 |  | R18A | 0.05109 | 1 | 0.9921 |
| D19A | 0.7621 | 1 | 0.8893 |  | D19A | 0.07715 | 2 | 0.9750 |
| K20A | 0.4664 | 2 | 0.7087 |  | K20A | 0.07462 | 1 | 0.9723 |
| S21A | 0.4493 | 2 | 0.9772 |  | S21A | 0.09939 | 1 | 0.9523 |
| V22A | 0.6798 | 2 | 0.9169 |  | V22A | 0.10190 | 1 | 0.9169 |
| T23A | 0.3620 | 1 | 0.9371 |  | T23A | 0.10530 | 1 | 0.9940 |
| I24A | 0.2925 | 2 | 0.2210 |  | I24A | 0.06930 | 2 | 0.8249 |
| Y25A | 0.9640 | 2 | 0.9919 |  | Y25A | 0.19100 | 2 | 0.9150 |
| L26A | 0.7955 | 1 | 0.9825 |  | L26A | 0.08093 | 1 | 0.9795 |
| G27A | 0.5497 | 1 | 0.9901 |  | G27A | 0.06795 | 1 | 0.9510 |
| K28A | 0.4649 | 1 | 0.9806 |  | K28A | 0.07916 | 1 | 0.9609 |
| R29A | 0.3938 | 2 | 0.9865 |  | R29A | 0.08106 | 1 | 0.7429 |
| D30A | 0.8562 | 2 | 0.9310 |  | D30A | 0.14690 | 2 | 0.9694 |
| Y31A | 0.4467 | 1 | 0.8411 |  | Y31A | 0.07137 | 1 | 0.9808 |
| Y32A | 0.7408 | 1 | 0.9404 |  | Y32A | 0.08474 | 1 | 0.9865 |
| D33A | 1.0000 | 1 | 0.9199 |  | D33A | 0.20340 | 2 | 0.9419 |
| H34A | 0.8303 | 1 | 0.9627 |  | H34A | 0.09519 | 1 | 0.9618 |
| V35A | 0.4522 | 2 | 0.9895 |  | V35A | 0.07716 | 2 | 0.9957 |
| E36A | 0.7652 | 1 | 0.9447 |  | E36A | 0.09319 | 1 | 0.8908 |
| R37A | 0.5648 | 1 | 0.9653 |  | R37A | 0.09114 | 1 | 0.9953 |
| V38A | 0.3957 | 1 | 0.9413 |  | V38A | 0.08623 | 1 | 0.9950 |
| E39A | 0.4686 | 1 | 0.9109 |  | E39A | 0.08638 | 1 | 0.9713 |
| P40A | 0.5263 | 1 | 0.9596 |  | P40A | 0.09295 | 1 | 0.9633 |
| V41A | 0.8138 | 1 | 0.8853 |  | V41A | 0.06235 | 1 | 0.9310 |
| D42A | 0.3573 | 1 | 0.9434 |  | D42A | 0.09144 | 1 | 0.7783 |
| G43A | 0.7465 | 2 | 0.9475 |  | G43A | 0.07777 | 2 | 0.9846 |
| V44A | 0.6139 | 2 | 0.8504 |  | V44A | 0.05502 | 2 | 0.9543 |
| V45A | 0.4469 | 2 | 0.9983 |  | V45A | 0.06701 | 2 | 0.9500 |
| L46A | 0.5222 | 2 | 0.9978 |  | L46A | 0.07173 | 2 | 0.9778 |
| V47A | 0.7378 | 2 | 0.7430 |  | V47A | 0.12190 | 2 | 0.9718 |
| D48A | 0.5608 | 2 | 0.9763 |  | D48A | 0.07061 | 2 | 0.9582 |
| P49A | 0.6405 | 2 | 0.9771 |  | P49A | 0.09037 | 2 | 0.9814 |
| E50A | 0.3716 | 2 | 0.5461 |  | E50A | 0.07931 | 2 | 0.9640 |
| L51A | 0.4627 | 2 | 0.9952 |  | L51A | 0.08669 | 2 | 0.9910 |
| V52A | 0.7587 | 1 | 0.9412 |  | V52A | 0.06219 | 1 | 0.9911 |
| K53A | 0.5996 | 1 | 0.9871 |  | K53A | 0.10390 | 1 | 0.8509 |
| G54A | 0.7343 | 1 | 0.9333 |  | G54A | 0.08433 | 1 | 0.9755 |
| K55A | 0.6482 | 1 | 0.9886 |  | K55A | 0.05807 | 1 | 0.9450 |
| R56A | 0.6463 | 1 | 0.9768 |  | R56A | 0.04869 | 1 | 0.9293 |
| V57A | 0.8198 | 1 | 0.9822 |  | V57A | 0.07263 | 1 | 0.9970 |
| Y58A | 0.5173 | 1 | 0.9653 |  | Y58A | 0.12010 | 1 | 0.9298 |
| V59A | 0.6059 | 1 | 0.9851 |  | V59A | 0.05866 | 1 | 0.9910 |
| S60A | 0.5622 | 2 | 0.9910 |  | S60A | 0.08221 | 1 | 0.8755 |
| L61A | 0.4309 | 1 | 0.9873 |  | L61A | 0.07984 | 1 | 0.9747 |
| T62A | 0.5386 | 2 | 0.9955 |  | T62A | 0.05232 | 1 | 0.9731 |
| C63A | 0.6134 | 2 | 0.9719 |  | C63A | 0.06839 | 1 | 0.9553 |
| A64G | 0.4394 | 2 | 0.9849 |  | A64G | 0.07754 | 1 | 0.9592 |
| F65A | 0.4463 | 3 | 0.9929 |  | F65A | 0.10360 | 1 | 0.9255 |
| R66A | 0.8681 | 3 | 0.9651 |  | R66A | 0.10130 | 2 | 0.9955 |
| Y67A | 0.6161 | 2 | 0.9765 |  | Y67A | 0.03651 | 1 | 0.9564 |
| G68A | 0.5630 | 2 | 0.9239 |  | G68A | 0.08554 | 3 | 0.9643 |
| Q69A | 0.4211 | 3 | 0.9888 |  | Q69A | 0.08131 | 2 | 0.9992 |
| E70A | 0.6140 | 4 | 0.9771 |  | E70A | 0.09928 | 3 | 0.9966 |
| D71A | 0.6151 | 3 | 0.9832 |  | D71A | 0.06320 | 2 | 0.9913 |
| I72A | 0.5131 | 2 | 0.9907 |  | I72A | 0.08812 | 1 | 0.9392 |
| D73A | 0.4618 | 4 | 0.9753 |  | D73A | 0.05936 | 3 | 0.9981 |
| V74A | 0.3254 | 3 | 0.9741 |  | V74A | 0.05742 | 2 | 0.9918 |
| M75A | 0.4052 | 3 | 0.9896 |  | M75A | 0.09830 | 2 | 0.9066 |
| G76A | 0.5698 | 2 | 0.3974 |  | G76A | 0.05749 | 1 | 0.9964 |
| L77A | 0.4633 | 4 | 0.9944 |  | L77A | 0.06832 | 3 | 0.9881 |
| S78A | 0.4779 | 1 | 0.9847 |  | S78A | 0.05533 | 1 | 0.8613 |
| F79A | 0.5443 | 2 | 0.8707 |  | F79A | 0.05629 | 1 | 0.9995 |
| R80A | 0.5719 | 2 | 0.9735 |  | R80A | 0.09130 | 2 | 0.9838 |
| R81A | 0.6746 | 3 | 0.8427 |  | R81A | 0.05678 | 2 | 0.9338 |
| D82A | 0.6579 | 2 | 0.9902 |  | D82A | 0.09136 | 2 | 0.9914 |
| L83A | 0.7645 | 1 | 0.9903 |  | L83A | 0.05120 | 1 | 0.9191 |
| Y84A | 0.6579 | 2 | 0.9809 |  | Y84A | 0.06027 | 1 | 0.9475 |
| F85A | 0.6679 | 2 | 0.6821 |  | F85A | 0.06636 | 1 | 0.9877 |
| S86A | 0.3109 | 2 | 0.9826 |  | S86A | 0.06734 | 1 | 0.9746 |
| Q87A | 0.5101 | 2 | 0.9776 |  | Q87A | 0.10950 | 1 | 0.9406 |
| V88A | 0.5849 | 4 | 0.9727 |  | V88A | 0.08277 | 3 | 0.9846 |
| Q89A | 0.5056 | 3 | 0.9007 |  | Q89A | 0.08277 | 3 | 0.9846 |
| V90A | 0.6396 | 3 | 0.9840 |  | V90A | 0.05393 | 3 | 0.9080 |
| F91A | 0.5894 | 4 | 0.9742 |  | F91A | 0.07761 | 4 | 0.9352 |
| P92A | 0.5766 | 3 | 0.9915 |  | P92A | 0.03188 | 2 | 0.9725 |
| P93A | 0.6377 | 4 | 0.9758 |  | P93A | 0.06212 | 3 | 0.9976 |
| V94A | 0.5842 | 4 | 0.9978 |  | V94A | 0.10990 | 3 | 0.9362 |
| G95A | 0.5831 | 2 | 0.9993 |  | G95A | 0.06466 | 1 | 0.9949 |
| A96G | 0.6117 | 2 | 0.9721 |  | A96G | 0.05974 | 1 | 0.9963 |
| S97A | 0.4573 | 3 | 0.9956 |  | S97A | 0.09039 | 2 | 0.9743 |
| G98A | 0.5282 | 2 | 0.9912 |  | G98A | 0.08193 | 1 | 0.9978 |
| A99G | 0.5030 | 1 | 0.9694 |  | A99G | 0.07583 | 1 | 0.9978 |
| T100A | 0.3544 | 1 | 0.9582 |  | T100A | 0.09200 | 1 | 0.9718 |
| T101A | 0.5837 | 1 | 0.9915 |  | T101A | 0.04638 | 1 | 0.9273 |
| R102A | 0.5139 | 2 | 0.9823 |  | R102A | 0.06226 | 1 | 0.7589 |
| L103A | 0.5100 | 2 | 0.9529 |  | L103A | 0.07688 | 2 | 0.9311 |
| Q104A | 0.6607 | 2 | 0.9880 |  | Q104A | 0.07180 | 2 | 0.9871 |
| E105A | 0.3897 | 2 | 0.8410 |  | E105A | 0.10150 | 2 | 0.8140 |
| S106A | 0.7485 | 1 | 0.9230 |  | S106A | 0.06404 | 1 | 0.9724 |
| L107A | 0.8063 | 1 | 0.9968 |  | L107A | 0.13630 | 2 | 0.8654 |
| I108A | 0.5167 | 3 | 0.9375 |  | I108A | 0.10810 | 2 | 0.7709 |
| K109A |  |  |  |  | K109A | 0.07781 | 1 | 0.9693 |
| K110A | 0.5112 | 2 | 0.9614 |  | K110A |  |  |  |
| L111A | 0.9531 | 2 | 0.9911 |  | L111A | 0.08559 | 1 | 0.8769 |
| G112A | 0.6350 | 1 | 0.7882 |  | G112A | 0.05555 | 1 | 0.9783 |
| A113G | 0.6633 | 2 | 0.9900 |  | A113G | 0.14450 | 2 | 0.7689 |
| N114A |  |  |  |  | N114A | 0.08539 | 1 | 0.9530 |
| T115A | 0.7504 | 1 | 0.7612 |  | T115A | 0.07780 | 1 | 0.9850 |
| Y116A | 0.5589 | 1 | 0.9831 |  | Y116A | 0.07102 | 1 | 0.9844 |
| P117A | 0.7098 | 1 | 0.9893 |  | P117A | 0.19690 | 1 | 0.9008 |
| F118A | 0.4193 | 1 | 0.9257 |  | F118A |  |  |  |
| L119A | 0.6853 | 1 | 0.9852 |  | L119A | 0.12070 | 1 | 0.9095 |
| L120A | 0.4307 | 1 | 0.7367 |  | L120A | 0.06849 | 1 | 0.9959 |
| T121A | 0.5957 | 1 | 0.9911 |  | T121A | 0.06359 | 1 | 0.9907 |
| F122A | 0.7800 | 1 | 0.9857 |  | F122A | 0.09607 | 1 | 0.9966 |
| P123A | 0.6501 | 1 | 0.9625 |  | P123A | 0.10300 | 1 | 0.9939 |
| D124A | 0.5802 | 1 | 0.9955 |  | D124A | 0.07773 | 1 | 0.9901 |
| Y125A | 0.5589 | 1 | 0.8885 |  | Y125A |  |  |  |
| L126A | 0.5167 | 1 | 0.7549 |  | L126A |  |  |  |
| P127A |  |  |  |  | P127A | 0.02662 | 1 | 0.4904 |
| C128A | 0.6372 | 1 | 0.9887 |  | C128A | 0.11380 | 1 | 0.6806 |
| S129A | 0.6532 | 1 | 0.6289 |  | S129A | 0.15000 | 1 | 0.8426 |
| V130A | 0.6116 | 1 | 0.9941 |  | V130A | 0.05068 | 1 | 0.7547 |
| M131A | 0.4426 | 1 | 0.7099 |  | M131A | 0.12060 | 1 | 0.4424 |
| L132A | 0.5563 | 1 | 0.8984 |  | L132A | 0.06474 | 1 | 0.9886 |
| Q133A | 0.3949 | 1 | 0.9027 |  | Q133A | 0.05933 | 1 | 0.9619 |
| P134A |  |  |  |  | P134A |  |  |  |
| A135G | 0.6993 | 1 | 0.9126 |  | A135G | 0.09610 | 1 | 0.9678 |
| P136A | 0.4234 | 1 | 0.7786 |  | P136A | 0.08771 | 1 | 0.9648 |
| Q137A | 0.4120 | 1 | 0.8292 |  | Q137A | 0.09044 | 1 | 0.9885 |
| D138A |  |  |  |  | D138A | 0.08616 | 1 | 0.9950 |
| V139A | 0.5117 | 1 | 0.9009 |  | V139A | 0.08936 | 1 | 0.9970 |
| G140A | 0.4972 | 1 | 0.9564 |  | G140A | 0.07038 | 1 | 0.9772 |
| K141A | 0.8690 | 1 | 0.9796 |  | K141A | 0.09105 | 1 | 0.9467 |
| S142A |  |  |  |  | S142A | 0.10070 | 1 | 0.9647 |
| C143A |  |  |  |  | C143A | 0.10420 | 1 | 0.9721 |
| G144A |  |  |  |  | G144A |  |  |  |
| V145A | 0.8758 | 1 | 0.7072 |  | V145A | 0.10690 | 1 | 0.9914 |
| D146A | 0.8282 | 1 | 0.9577 |  | D146A | 0.11390 | 1 | 0.8508 |
| F147A | 0.6024 | 1 | 0.9065 |  | F147A | 0.09670 | 1 | 0.9687 |
| E148A |  |  |  |  | E148A | 0.08897 | 1 | 0.9023 |
| I149A |  |  |  |  | I149A | 0.10620 | 1 | 0.9709 |
| K150A | 0.8346 | 1 | 0.9401 |  | K150A | 0.05089 | 1 | 0.9953 |
| A151G |  |  |  |  | A151G | 0.10530 | 1 | 0.4994 |
| F152A |  |  |  |  | F152A | 0.06378 | 1 | 0.8271 |
| A153G | 0.6682 | 1 | 0.8089 |  | A153G | 0.03594 | 1 | 0.5655 |
| T154A |  |  |  |  | T154A |  |  |  |
| H155A | 0.3800 | 1 | 0.9783 |  | H155A | 0.07181 | 1 | 0.9105 |
| S156A |  |  |  |  | S156A | 0.06953 | 1 | 0.9829 |
| T157A |  |  |  |  | T157A |  |  |  |
| D158A | 0.4005 | 1 | 0.9628 |  | D158A | 0.06953 | 1 | 0.9829 |
| V159A | 0.6778 | 1 | 0.9519 |  | V159A | 0.09389 | 1 | 0.8905 |
| E160A | 0.5301 | 1 | 0.6685 |  | E160A | 0.06727 | 1 | 0.9887 |
| E161A | 0.3539 | 1 | 0.9726 |  | E161A | 0.06512 | 1 | 0.9895 |
| D162A | 0.4124 | 1 | 0.9933 |  | D162A | 0.10020 | 1 | 0.9844 |
| K163A | 0.4432 | 1 | 0.9360 |  | K163A | 0.06138 | 1 | 0.9597 |
| I164A | 0.4953 | 1 | 0.5361 |  | I164A | 0.07400 | 1 | 0.9908 |
| P165A | 0.3473 | 1 | 0.9932 |  | P165A | 0.09077 | 1 | 0.9791 |
| K166A | 0.6928 | 2 | 0.9269 |  | K166A | 0.05972 | 1 | 0.9355 |
| K167A | 0.6040 | 1 | 0.7303 |  | K167A | 0.07145 | 1 | 0.9806 |
| S168A | 0.5520 | 1 | 0.7893 |  | S168A | 0.09372 | 1 | 0.9934 |
| S169A | 0.4345 | 1 | 0.9890 |  | S169A | 0.04153 | 1 | 0.9697 |
| V170A | 0.7231 | 1 | 0.7776 |  | V170A | 0.13630 | 1 | 0.8875 |
| R171A | 0.9405 | 1 | 0.9348 |  | R171A | 0.05481 | 1 | 0.9846 |
| L172A |  |  |  |  | L172A | 0.08082 | 1 | 0.9603 |
| L173A |  |  |  |  | L173A |  |  |  |
| I174A | 0.5631 | 1 | 0.9693 |  | I174A | 0.07441 | 1 | 0.9911 |
| R175A | 1.2890 | 1 | 0.5058 |  | R175A | 0.13390 | 1 | 0.9475 |
| K176A | 0.7195 | 1 | 0.9340 |  | K176A | 0.12660 | 1 | 0.9311 |
| V177A | 0.6477 | 1 | 0.9477 |  | V177A | 0.08469 | 1 | 0.9214 |
| Q178A | 0.6092 | 1 | 0.9757 |  | Q178A | 0.07103 | 1 | 0.9908 |
| H179A | 0.4841 | 1 | 0.9891 |  | H179A | 0.10590 | 1 | 0.9353 |
| A180G | 0.5979 | 1 | 0.9367 |  | A180G | 0.08570 | 1 | 0.9362 |
| P181A | 0.5142 | 1 | 0.9891 |  | P181A | 0.04913 | 1 | 0.9781 |
| R182A | 0.7688 | 1 | 0.9931 |  | R182A | 0.06663 | 1 | 0.9903 |
| D183A | 0.6203 | 1 | 0.9795 |  | D183A | 0.08646 | 1 | 0.9849 |
| M184A | 0.2805 | 1 | 0.9142 |  | M184A |  |  |  |
| G185A | 0.6764 | 1 | 0.9392 |  | G185A |  |  |  |
| P186A | 0.4926 | 1 | 0.9794 |  | P186A |  |  |  |
| Q187A | 0.4808 | 1 | 0.9760 |  | Q187A |  |  |  |
| P188A | 0.3065 | 1 | 0.9785 |  | P188A |  |  |  |
| R189A | 0.4516 | 1 | 0.9892 |  | R189A |  |  |  |
| A190G | 0.4246 | 1 | 0.9817 |  | A190G |  |  |  |
| E191A | 0.5118 | 1 | 0.8555 |  | E191A |  |  |  |
| A192G | 0.4562 | 1 | 0.9669 |  | A192G | 0.10800 | 1 | 0.9909 |
| S193A | 0.6678 | 1 | 0.8943 |  | S193A | 0.09208 | 1 | 1.0000 |
| W194A | 0.2863 | 1 | 0.9898 |  | W194A |  |  |  |
| P195A | 0.3913 | 1 | 0.9279 |  | P195A | 0.05765 | 1 | 0.9313 |
| F196A | 0.4807 | 1 | 0.9959 |  | F196A | 0.05985 | 1 | 0.8099 |
| F197A | 0.4337 | 2 | 0.9758 |  | F197A | 0.09280 | 2 | 0.9981 |
| M198A | 0.3799 | 2 | 0.9960 |  | M198A | 0.07817 | 2 | 0.9964 |
| S199A | 0.3200 | 2 | 0.9957 |  | S199A | 0.06870 | 2 | 0.9948 |
| D200A | 0.5862 | 2 | 0.6363 |  | D200A | 0.08787 | 2 | 1.0000 |
| K201A |  |  |  |  | K201A | 0.09015 | 1 | 0.9854 |
| P202A | 0.3770 | 1 | 0.6111 |  | P202A | 0.08534 | 1 | 0.9352 |
| L203A | 0.2938 | 1 | 0.9679 |  | L203A | 0.06782 | 1 | 0.9714 |
| R204A |  |  |  |  | R204A | 0.07841 | 2 | 0.9776 |
| L205A |  |  |  |  | L205A | 0.09016 | 2 | 0.9964 |
| A206G | 0.5389 | 1 | 0.9661 |  | A206G | 0.06282 | 2 | 0.9939 |
| V207A | 0.4904 | 1 | 0.9857 |  | V207A | 0.06282 | 2 | 0.9939 |
| S208A | 0.5754 | 1 | 0.9210 |  | S208A | 0.06948 | 2 | 0.9985 |
| L209A | 0.7714 | 1 | 0.9268 |  | L209A | 0.06669 | 2 | 0.9812 |
| S210A | 0.5756 | 1 | 0.9931 |  | S210A | 0.07506 | 2 | 0.9912 |
| K211A | 0.6697 | 1 | 0.9773 |  | K211A | 0.06366 | 2 | 0.9826 |
| E212A | 0.4552 | 1 | 0.9872 |  | E212A | 0.11940 | 2 | 0.9748 |
| I213A |  |  |  |  | I213A | 0.06876 | 2 | 0.9945 |
| Y214A | 0.4486 | 1 | 0.9626 |  | Y214A | 0.09413 | 2 | 0.9410 |
| Y215A | 0.4402 | 1 | 0.9573 |  | Y215A | 0.07959 | 2 | 0.9767 |
| H216A | 0.3980 | 1 | 0.9479 |  | H216A | 0.08250 | 2 | 0.9646 |
| G217A | 0.6242 | 1 | 0.9945 |  | G217A | 0.06160 | 2 | 0.9560 |
| E218A | 0.5996 | 1 | 0.9869 |  | E218A | 0.06868 | 2 | 0.9589 |
| P219A | 0.5198 | 1 | 0.9724 |  | P219A | 0.07060 | 2 | 0.9918 |
| I220A | 0.6817 | 1 | 0.8987 |  | I220A | 0.05931 | 2 | 0.9583 |
| P221A | 0.6146 | 1 | 0.9746 |  | P221A | 0.08209 | 2 | 0.9956 |
| V222A | 0.6468 | 1 | 0.9812 |  | V222A | 0.07489 | 2 | 0.9706 |
| T223A | 0.6107 | 1 | 0.9453 |  | T223A | 0.07763 | 2 | 0.9989 |
| V224A | 0.7049 | 1 | 0.9694 |  | V224A | 0.06582 | 2 | 0.7813 |
| A225G | 0.4515 | 1 | 0.5058 |  | A225G | 0.08881 | 1 | 0.9895 |
| V226A | 0.5051 | 1 | 0.9304 |  | V226A | 0.08535 | 1 | 0.9876 |
| T227A | 0.5107 | 1 | 0.9702 |  | T227A | 0.08107 | 1 | 0.9985 |
| N228A | 0.5057 | 1 | 0.9957 |  | N228A | 0.07760 | 1 | 0.9738 |
| S229A | 0.6560 | 1 | 0.9866 |  | S229A | 0.08201 | 1 | 0.9979 |
| T230A | 0.6137 | 1 | 0.9987 |  | T230A | 0.08204 | 1 | 0.9953 |
| E231A | 0.4538 | 1 | 0.9093 |  | E231A | 0.08443 | 2 | 0.9991 |
| K232A | 0.5075 | 1 | 0.9879 |  | K232A | 0.06484 | 2 | 0.9770 |
| T233A | 0.4195 | 1 | 0.9986 |  | T233A | 0.08108 | 2 | 0.9715 |
| V234A | 0.4926 | 2 | 0.9750 |  | V234A | 0.13820 | 3 | 0.9898 |
| K235A | 0.6221 | 2 | 0.9393 |  | K235A | 0.08352 | 2 | 0.9925 |
| K236A | 0.6254 | 1 | 0.4580 |  | K236A | 0.08350 | 1 | 0.8472 |
| I237A | 0.5481 | 2 | 0.9978 |  | I237A | 0.07654 | 2 | 0.8889 |
| K238A | 0.7217 | 1 | 0.9644 |  | K238A |  |  |  |
| V239A | 0.6597 | 2 | 0.9824 |  | V239A | 0.07230 | 2 | 0.9885 |
| L240A | 0.4538 | 1 | 0.9413 |  | L240A | 0.10410 | 1 | 0.9617 |
| V241A | 0.5000 | 1 | 0.6667 |  | V241A |  |  |  |
| E242A | 0.5718 | 1 | 0.9468 |  | E242A | 0.09492 | 1 | 0.9955 |
| Q243A | 0.4177 | 1 | 0.5904 |  | Q243A | 0.07917 | 1 | 0.9958 |
| V244A | 0.4577 | 1 | 0.9845 |  | V244A | 0.07649 | 1 | 0.9992 |
| T245A | 0.6487 | 1 | 0.9230 |  | T245A | 0.09258 | 1 | 0.9992 |
| N246A | 0.5235 | 1 | 0.9687 |  | N246A | 0.06522 | 1 | 0.9611 |
| V247A |  |  |  |  | V247A |  |  |  |
| V248A | 0.5177 | 1 | 0.9947 |  | V248A | 0.07825 | 1 | 0.9861 |
| L249A | 0.4023 | 2 | 0.9653 |  | L249A | 0.08107 | 1 | 0.9537 |
| Y250A | 0.4484 | 2 | 0.9913 |  | Y250A | 0.09015 | 1 | 0.9631 |
| S251A | 0.4250 | 1 | 0.9846 |  | S251A | 0.07605 | 1 | 0.9973 |
| S252A | 0.5160 | 2 | 0.9987 |  | S252A | 0.06120 | 1 | 0.9892 |
| D253A | 0.5612 | 1 | 0.9955 |  | D253A | 0.08937 | 1 | 0.9882 |
| V254A | 0.4538 | 1 | 0.9874 |  | V254A |  |  |  |
| Y255A |  |  |  |  | Y255A | 0.06277 | 1 | 0.9776 |
| I256A |  |  |  |  | I256A |  |  |  |
| K257A | 0.5722 | 1 | 0.9947 |  | K257A | 0.09493 | 1 | 0.9991 |
| T258A | 0.4879 | 1 | 0.8730 |  | T258A | 0.05094 | 1 | 0.9340 |
| V259A | 0.4938 | 1 | 0.9861 |  | V259A | 0.06847 | 1 | 0.9955 |
| A260G | 0.7023 | 1 | 0.9369 |  | A260G | 0.09526 | 1 | 0.9828 |
| A261G | 0.5902 | 1 | 0.9989 |  | A261G | 0.08280 | 1 | 0.9827 |
| E262A | 0.5409 | 1 | 0.9046 |  | E262A | 0.08275 | 1 | 0.8938 |
| E263A | 0.4943 | 1 | 0.9782 |  | E263A | 0.05814 | 1 | 0.9221 |
| A264G | 0.4580 | 1 | 0.9595 |  | A264G | 0.04952 | 1 | 0.9805 |
| Q265A | 0.5536 | 1 | 0.9815 |  | Q265A | 0.06197 | 1 | 0.9480 |
| E266A | 0.4685 | 1 | 0.9891 |  | E266A | 0.07884 | 1 | 0.9929 |
| K267A | 0.5014 | 2 | 0.9938 |  | K267A | 0.05915 | 2 | 0.8077 |
| V268A | 0.7580 | 2 | 0.9528 |  | V268A | 0.06156 | 2 | 0.8958 |
| P269A | 0.9470 | 1 | 0.9616 |  | P269A | 0.07036 | 1 | 0.9920 |
| P270A | 0.7099 | 1 | 0.9249 |  | P270A | 0.09318 | 1 | 0.9954 |
| N271A | 0.6972 | 1 | 0.9463 |  | N271A | 0.06064 | 1 | 0.9267 |
| S272A | 0.8690 | 1 | 0.9914 |  | S272A | 0.06744 | 1 | 0.9473 |
| S273A | 0.8386 | 1 | 0.8324 |  | S273A | 0.05510 | 1 | 0.9387 |
| L274A | 0.8316 | 1 | 0.9455 |  | L274A | 0.05386 | 1 | 0.9850 |
| T275A |  |  |  |  | T275A | 0.04170 | 1 | 0.9697 |
| K276A | 0.5866 | 1 | 0.9109 |  | K276A | 0.07094 | 1 | 0.9953 |
| T277A | 0.4385 | 1 | 0.9936 |  | T277A | 0.04828 | 1 | 0.9960 |
| L278A | 0.9207 | 1 | 0.9416 |  | L278A | 0.10170 | 1 | 0.9441 |
| T279A | 0.5995 | 1 | 0.9804 |  | T279A | 0.07934 | 1 | 0.9974 |
| L280A | 0.5039 | 1 | 0.9416 |  | L280A | 0.08095 | 1 | 0.9961 |
| V281A | 0.5305 | 1 | 0.9907 |  | V281A | 0.04962 | 1 | 0.9948 |
| P282A | 0.4765 | 1 | 0.9801 |  | P282A | 0.08019 | 1 | 0.9555 |
| L283A | 0.5880 | 1 | 0.9807 |  | L283A | 0.08074 | 1 | 0.9809 |
| L284A | 0.8278 | 1 | 0.9705 |  | L284A | 0.11190 | 1 | 0.9872 |
| A285G | 0.4581 | 1 | 0.9966 |  | A285G | 0.04216 | 1 | 0.8955 |
| N286A | 0.2214 | 1 | 0.9008 |  | N286A | 0.07610 | 1 | 0.9455 |
| N287A | 0.4153 | 1 | 0.9960 |  | N287A | 0.04431 | 1 | 0.9758 |
| R288A |  |  |  |  | R288A |  |  |  |
| E289A |  |  |  |  | E289A |  |  |  |
| R290A |  |  |  |  | R290A |  |  |  |
| R291A |  |  |  |  | R291A |  |  |  |
| G292A | 0.4535 | 1 | 0.6170 |  | G292A | 0.09568 | 1 | 0.9548 |
| I293A | 0.3791 | 1 | 0.7211 |  | I293A | 0.08924 | 1 | 0.9124 |
| A294G | 0.5465 | 1 | 0.9699 |  | A294G | 0.06408 | 1 | 0.9751 |
| L295A | 0.3450 | 1 | 0.8917 |  | L295A | 0.07309 | 1 | 0.9803 |
| D296A | 0.7410 | 2 | 0.8861 |  | D296A | 0.08550 | 2 | 0.9801 |
| G297A | 0.6776 | 2 | 0.8332 |  | G297A | 0.10450 | 2 | 0.9654 |
| K298A | 0.5162 | 1 | 0.5905 |  | K298A | 0.07737 | 2 | 0.9996 |
| I299A | 0.6291 | 2 | 0.9531 |  | I299A | 0.08459 | 2 | 0.9822 |
| K300A | 0.5017 | 3 | 0.9546 |  | K300A | 0.06948 | 2 | 0.9886 |
| H301A | 0.3467 | 2 | 0.8543 |  | H301A | 0.11980 | 2 | 0.9813 |
| E302A | 0.5733 | 2 | 0.9873 |  | E302A | 0.07799 | 2 | 0.9994 |
| D303A | 0.7479 | 2 | 0.7121 |  | D303A | 0.12300 | 2 | 0.6836 |
| T304A | 0.9266 | 2 | 0.8625 |  | T304A | 0.09814 | 2 | 0.8058 |
| N305A | 0.4770 | 2 | 0.9880 |  | N305A | 0.05495 | 2 | 0.9824 |
| L306A | 0.5212 | 2 | 0.9834 |  | L306A | 0.08722 | 2 | 0.9922 |
| A307G |  |  |  |  | A307G |  |  |  |
| S308A | 0.3284 | 2 | 0.9944 |  | S308A | 0.06402 | 1 | 0.8761 |
| S309A | 0.4574 | 1 | 0.8559 |  | S309A | 0.08456 | 1 | 0.9908 |
| T310A | 0.4022 | 1 | 0.8221 |  | T310A | 0.11850 | 1 | 0.9991 |
| I311A | 0.2849 | 1 | 0.9221 |  | I311A | 0.08669 | 1 | 0.9978 |
| I312A | 0.2618 | 1 | 0.9472 |  | I312A | 0.06815 | 1 | 0.9980 |
| K313A |  |  |  |  | K313A |  |  |  |
| E314A |  |  |  |  | E314A |  |  |  |
| G315A | 0.3049 | 1 | 0.9776 |  | G315A | 0.09040 | 1 | 0.9959 |
| I316A | 0.4704 | 1 | 0.9972 |  | I316A | 0.08360 | 1 | 0.9919 |
| D317A | 0.4413 | 1 | 0.9052 |  | D317A | 0.11470 | 1 | 0.9200 |
| K318A | 0.6808 | 1 | 0.8451 |  | K318A | 0.13580 | 1 | 0.9932 |
| T319A | 0.5695 | 1 | 0.9855 |  | T319A | 0.12770 | 1 | 0.9118 |
| V320A |  |  |  |  | V320A |  |  |  |
| M321A | 0.4924 | 1 | 0.8807 |  | M321A | 0.12650 | 1 | 0.7530 |
| G322A |  |  |  |  | G322A |  |  |  |
| I323A | 0.4023 | 1 | 0.9003 |  | I323A | 0.20950 | 1 | 0.9490 |
| L324A | 0.4332 | 1 | 0.9349 |  | L324A | 0.11640 | 1 | 0.8500 |
| V325A |  |  |  |  | V325A |  |  |  |
| S326A | 0.5387 | 1 | 0.7558 |  | S326A | 0.13680 | 1 | 0.9343 |
| Y327A |  |  |  |  | Y327A |  |  |  |
| Q328A | 0.5585 | 1 | 0.9751 |  | Q328A | 0.10910 | 1 | 0.9721 |
| I329A | 0.6262 | 2 | 0.9895 |  | I329A | 0.11090 | 2 | 0.9733 |
| K330A | 0.5789 | 2 | 0.9901 |  | K330A | 0.11800 | 2 | 0.9785 |
| V331A | 0.5246 | 2 | 0.9753 |  | V331A | 0.12780 | 2 | 0.9848 |
| K332A | 0.5759 | 2 | 0.9794 |  | K332A | 0.08185 | 2 | 0.9095 |
| L333A | 0.3330 | 1 | 0.8888 |  | L333A | 0.13660 | 1 | 0.9860 |
| T334A | 0.4458 | 1 | 0.9980 |  | T334A | 0.13020 | 1 | 0.9640 |
| V335A | 0.5687 | 1 | 0.8797 |  | V335A | 0.13070 | 1 | 0.9897 |
| S336A | 0.5059 | 2 | 0.9984 |  | S336A | 0.10370 | 2 | 0.9668 |
| G337A | 0.3824 | 2 | 0.9851 |  | G337A | 0.18860 | 2 | 0.9333 |
| L338A | 0.4380 | 2 | 0.9922 |  | L338A | 0.08307 | 2 | 0.9681 |
| L339A | 0.3003 | 2 | 0.9603 |  | L339A | 0.12200 | 2 | 0.9786 |
| G340A | 0.4792 | 2 | 0.9782 |  | G340A | 0.10430 | 2 | 0.9902 |
| E341A | 0.5549 | 2 | 0.9933 |  | E341A | 0.11820 | 2 | 0.9772 |
| L342A | 0.2911 | 2 | 0.9938 |  | L342A | 0.19440 | 2 | 0.9356 |
| T343A |  |  |  |  | T343A |  |  |  |
| S344A |  |  |  |  | S344A |  |  |  |
| S345A | 0.3659 | 1 | 0.9973 |  | S345A | 0.10430 | 1 | 0.9900 |
| S346A |  |  |  |  | S346A |  |  |  |
| V347A | 0.4438 | 1 | 0.8705 |  | V347A | 0.09342 | 1 | 0.9951 |
| A348G | 0.4143 | 1 | 0.9376 |  | A348G | 0.10650 | 1 | 0.9585 |
| T349A | 0.2901 | 1 | 0.9567 |  | T349A | 0.05926 | 1 | 0.9623 |
| E350A | 0.5058 | 1 | 0.8627 |  | E350A | 0.06888 | 1 | 0.9976 |
| V351A | 0.4439 | 1 | 0.9842 |  | V351A | 0.07790 | 1 | 0.9755 |
| P352A | 0.5917 | 1 | 0.8638 |  | P352A | 0.08106 | 1 | 0.9793 |
| F353A | 0.5014 | 1 | 0.9561 |  | F353A | 0.07620 | 1 | 0.8939 |
| R354A | 0.3221 | 1 | 0.9853 |  | R354A | 0.10780 | 1 | 0.7441 |
| L355A | 0.8753 | 1 | 0.9730 |  | L355A | 0.17410 | 1 | 0.8417 |
| M356A | 0.5760 | 1 | 0.9574 |  | M356A | 0.10590 | 1 | 0.9900 |
| H357A | 0.9258 | 1 | 0.9107 |  | H357A | 0.05041 | 1 | 0.2139 |
| P358A | 0.6268 | 1 | 0.8947 |  | P358A | 0.06925 | 1 | 0.9938 |
| Q359A |  |  |  |  | Q359A | 0.06509 | 1 | 0.9898 |
| P360A | 0.4420 | 1 | 0.9932 |  | P360A | 0.06498 | 1 | 0.9899 |
| E361A |  |  |  |  | E361A | 0.08478 | 1 | 0.9003 |
| D362A | 0.6755 | 1 | 0.9761 |  | D362A | 0.09912 | 1 | 0.9878 |
| P363A |  |  |  |  | P363A |  |  |  |
| D364A |  |  |  |  | D364A | 0.07676 | 1 | 0.9969 |
| T365A |  |  |  |  | T365A | 0.09947 | 1 | 0.8619 |
| A366G | 0.6393 | 1 | 0.9185 |  | A366G |  |  |  |
| K367A | 0.3609 | 1 | 0.9954 |  | K367A | 0.05902 | 1 | 0.9800 |
| E368A |  |  |  |  | E368A |  |  |  |
| S369A |  |  |  |  | S369A | 0.23880 | 1 | 0.9034 |
| F370A | 0.6946 | 1 | 0.9394 |  | F370A | 0.11570 | 1 | 0.9425 |
| Q371A | 0.4439 | 1 | 0.9061 |  | Q371A |  |  |  |
| D372A |  |  |  |  | D372A | 0.07578 | 1 | 0.9523 |
| E373A | 0.5312 | 1 | 0.8084 |  | E373A | 0.04577 | 1 | 0.9127 |
| N374A | 0.5383 | 1 | 0.9988 |  | N374A | 0.14970 | 2 | 0.9323 |
| F375A | 0.8570 | 2 | 0.9616 |  | F375A | 0.21080 | 3 | 0.9813 |
| V376A |  |  |  |  | V376A | 0.12340 | 1 | 0.8399 |
| F377A | 0.8094 | 2 | 0.9414 |  | F377A | 0.22830 | 2 | 0.8756 |
| E378A | 0.5311 | 2 | 0.9852 |  | E378A | 0.13380 | 2 | 0.9781 |
| E379A |  |  |  |  | E379A |  |  |  |
| F380A | 1.0200 | 1 | 0.9890 |  | F380A | 0.16230 | 1 | 0.9469 |
| A381G | 1.3540 | 1 | 0.8560 |  | A381G |  |  |  |
| R382A | 1.1920 | 1 | 0.9050 |  | R382A | 0.16970 | 1 | 0.9676 |
| Q383A | 0.3411 | 1 | 0.9937 |  | Q383A | 0.08245 | 1 | 0.9728 |
| N384A |  |  |  |  | N384A |  |  |  |
| L385A | 0.4161 | 1 | 0.9828 |  | L385A | 0.07101 | 1 | 0.9994 |
| K386A |  |  |  |  | K386A |  |  |  |
| D387A | 0.7390 | 1 | 0.8935 |  | D387A | 0.07970 | 1 | 0.9133 |
| A388G |  |  |  |  | A388G | 0.07455 | 1 | 0.9981 |
| G389A | 0.7384 | 1 | 0.9689 |  | G389A | 0.08383 | 1 | 0.9878 |
| E390A |  |  |  |  | E390A |  |  |  |
| Y391A | 0.4560 | 1 | 0.9595 |  | Y391A | 0.07830 | 1 | 0.9953 |
| K392A | 0.7574 | 1 | 0.9486 |  | K392A | 0.07556 | 1 | 0.9639 |
| E393A | 0.4265 | 1 | 0.8209 |  | E393A | 0.08889 | 1 | 0.9979 |
| E394A |  |  |  |  | E394A |  |  |  |
| K395A | 0.5749 | 1 | 0.9763 |  | K395A |  |  |  |
| T396A |  |  |  |  | T396A | 0.08481 | 1 | 0.9943 |
| D397A | 0.6346 | 1 | 0.9241 |  | D397A | 0.06987 | 1 | 0.9640 |
| Q398A | 0.5384 | 1 | 0.9549 |  | Q398A | 0.09460 | 1 | 0.9966 |
| E399A | 0.5247 | 1 | 0.9638 |  | E399A | 0.09131 | 1 | 0.9962 |
| A400G | 0.6486 | 1 | 0.9992 |  | A400G | 0.07951 | 1 | 0.9897 |
| A401G | 0.5836 | 1 | 0.8738 |  | A401G | 0.08668 | 1 | 0.9937 |
| M402A | 0.3470 | 1 | 0.9823 |  | M402A | 0.07855 | 1 | 0.9756 |
| D403A | 0.5764 | 1 | 0.9528 |  | D403A | 0.09436 | 1 | 0.9656 |
| deltaC | 0.8230 | 2 | 0.9102 |  | deltaC | 0.29930 | 2 | 0.8484 |
| **WT** | **0.5911** | **40** | **0.9579** |  | **WT** | **0.07566** | **40** | **0.9988** |

**Supplementary Table 1:** List of IC_50_ values arrestin mutant binding to Meta II-P (red) and Ops-P (blue). IC_50_ values have been obtained from fitting of a sigmoidal response curve to 8 different salt concentrations with the quality of fit indicated as R^2^. A selection of functionally important residues has been measured multiple times and the values averaged. Missing mutations were removed from analysis either because they did not express (as indicated by in gel-fluorescence of the mCherry fluorescence marker) or expression was too low to obtain a reliable signal.
